# Re-purposing 16S rRNA gene sequence data from within case paired tumor biopsy and tumor-adjacent biopsy or fecal samples to identify microbial markers for colorectal cancer

**DOI:** 10.1371/journal.pone.0207002

**Published:** 2018-11-09

**Authors:** Manasi S. Shah, Todd DeSantis, Jose-Miguel Yamal, Tiffany Weir, Elizabeth P. Ryan, Julia L. Cope, Emily B. Hollister

**Affiliations:** 1 University of Texas School of Public Health, Houston, TX, United States of America; 2 Informatics, Second Genome, Inc, South San Francisco, CA, United States of America; 3 Department of Pathology & Immunology, Baylor College of Medicine, Houston, TX, United States of America; 4 Texas Children’s Microbiome Center, Department of Pathology, Texas Children’s Hospital, Houston, TX, United States of America; 5 Department of Food Science and Human Nutrition, Colorado State University, Fort Collins, CO, United States of America; 6 Department of Environmental and Radiological Health Sciences, Colorado State University/Colorado School of Public Health, Fort Collins, CO, United States of America; 7 Diversigen, Inc, Houston, TX, United States of America; Ohio State University Wexner Medical Center, UNITED STATES

## Abstract

Microbes colonizing colorectal cancer (CRC) tumors have the potential to affect disease, and vice-versa. The manner in which they differ from microbes in physically adjacent tissue or stool within the case in terms of both, taxonomy and biological activity remains unclear. In this study, we systematically analyzed previously published 16S rRNA sequence data from CRC patients with matched tumor:tumor-adjacent biopsies (n = 294 pairs, n = 588 biospecimens) and matched tumor biopsy:fecal pairs (n = 42 pairs, n = 84 biospecimens). Procrustes analyses, random effects regression, random forest (RF) modeling, and inferred functional pathway analyses were conducted to assess community similarity and microbial diversity across heterogeneous patient groups and studies. Our results corroborate previously reported association of increased *Fusobacterium* with tumor biopsies. *Parvimonas* and *Streptococcus* abundances were also elevated while *Faecalibacterium* and Ruminococcaceae abundances decreased in tumors relative to tumor-adjacent biopsies and stool samples from the same case. With the exception of these limited taxa, the majority of findings from individual studies were not confirmed by other 16S rRNA gene-based datasets. RF models comparing tumor and tumor-adjacent specimens yielded an area under curve (AUC) of 64.3%, and models of tumor biopsies versus fecal specimens exhibited an AUC of 82.5%. Although some taxa were shared between fecal and tumor samples, their relative abundances varied substantially. Inferred functional analysis identified potential differences in branched amino acid and lipid metabolism. Microbial markers that reliably occur in tumor tissue can have implications for microbiome based and microbiome targeting therapeutics for CRC.

## Introduction

Increasing evidence suggests that the gastrointestinal microbiome, both luminal (*i*.*e*., fecal) and mucosal (i.e biopsy based), may be involved in mediating the onset and/or progression of colorectal cancer (CRC) [1–4]. Fecal microbiota can affect tumor development via energy harvest and the production of metabolites, such as secondary bile acids. Lithocholic and deoxycholic acid, for example, are enriched in the fecal contents of CRC patients and known to activate the NF-kB signaling pathway, which can promote resistance to chemotherapy in colonic epithelial cells [5]. Mucosal microbiota can influence carcinogenesis mechanistically by modulation of the host immune system (*e*.*g*., production of pro-inflammatory cytokines, which interact with Goblet and Paneth cells and compromise barrier function) and/or the innate immune system (*e*.*g*., tumor activation via NF-kB and STAT3 signaling pathways, including Toll-like receptor 4 (TLR4) activation and up regulation of the PTGS2 and EGFR signaling pathway) [6, 7]. *Fusobacterium nucleatum* expresses the FadA virulence factor, correlates withWnt pathway activation in colorectal carcinoma cells, and has been shown to induce resistance to chemotherapy in vitro by activating the autophagy pathway [8, 9]. Other in-vitro studies have shown that *Bacteroides fragilis* produces a genotoxin and is known to activate the Wnt and NFKB pathways [10] and members of *Escherchia coli* phylogroup B2 produce cytolethal distending toxin and have been shown to induce DNA damage and influence genome stability in mice [11].

Despite recognition of these key taxa, considerable cohort to cohort differences have been reported among mucosal microbial taxa from CRC patients [2, 12–14]. This may be attributed to clinical differences among patients and cohorts, as well as technical differences among experimental protocols, including the physical location(s) from which samples are collected. The spatial organization of bacteria along the gastrointestinal tract is highly variable and contingent upon nutrient availability, physical characteristics like oxygen gradients, pH, and host immunomodulation [15]. In addition, some studies found fecal populations to be less representative of disease-associated dysbiosis than their mucosal counterparts [6, 16]. Evaluating on-tumor versus off-tumor microbial communities and mucosal versus fecal taxonomic disparities in the context of CRC has been hindered by the limited number of studies that have examined differences in both the mucosal (both tumor and tumor-adjacent tissue) and fecal microbiota within the same colorectal cancer cases [6, 17–19]. To this end, aims for our study were three-fold. We sought to mine publicly available CRC microbiome datasets 1) to evaluate the degree to which tumor-associated microbial communities were consistent with one another across studies (vs. non-affected tissues) 2) to impute mechanistic pathways through which mucosal markers might operate and 3) to determine the degree to which fecal and mucosal microbial communities overlap with one another. Although we and others have shown that fecal microbes have strong potential to serve in a diagnostic capacity [4, 20, 21], the degree to which these microbes reflect disease biology and provide mechanistic insight with respect to disease onset and development are unclear. The potential disconnect between mucosal and fecal microbial communities was a motivating factor for this study.

While we were preparing this study for submission, Sze et al. published a similar study aggregating fecal and tumor tissue microbial data from colorectal cancer cases. Findings from Sze et al. were concordant with our original fecal sample-based analysis of microbial markers and found a similar set of markers such as enrichment of *Fusobacterium and Parvimonas* and depletion of *Ruminococcus* in fecal CRC samples relative to controls [4, 21]. Sze et al. also compared microbial taxa in both tumor and/or adenoma versus pathologically healthy tissue either within the case or from external healthy controls. However, in the study presented here, we specifically focused on tumor tissue, adjacent pathologically tumor-free tissue, or fecal samples collected from the *same* colorectal cancer case to control for confounding factors such as host genetics, expression and immune response, each of which are known to strongly affect composition of microbial communities. For this comparison, our study also includes five additional cohorts in the final analysis [2, 14, 22–24], resulting in 588 paired (matched samples) versus the 381 matched tumor:tumor-adjacent CRC biopsy samples, making it a more comprehensive analysis representing greater variability (and noise) in the available data.

## Methods

### Bioinformatics analysis

A systematic search was conducted to identify reports on human-based studies of the colorectal cancer microbiome that had been published within the last ten years. This was accomplished using Pubmed’s advanced search feature as follows: ((((((((((bacterial microbiome OR gut microbiome OR microbiota OR microbial)) AND (fecal OR mucosal OR biopsy OR luminal OR colonic or tumor or tissue or feces)) AND (colorectal cancer[Title] OR colon cancer[Title] OR colorectal adenoma[Title] OR adenomatous polyp[Title] or colorectal carcinoma[Title])) AND ("2006/01/01"[PDAT]: "2016/04/01"[PDAT])) AND humans[MeSH Terms]) NOT review[Publication Type]) AND Humans[Mesh])). This search returned 119 results. All studies included in the final analysis: (i) used the 454 or Illumina sequencing platforms for sequencing of 16S rRNA gene amplicons from biopsy specimens; (ii) included histologically-confirmed CRC tumor:tumor-adjacent biopsy or tumor biopsy:fecal samples from same CRC case; and (iii) made sequence and associated metadata available in the public realm (or shared by authors on or before April 1^st^ 2016).

A total of 14 studies satisfied the inclusion criteria described above (Table 1), 11 of which provided access to their raw data in public repositories or upon request [2, 6, 12–14, 17–19, 23–26] (S1 Table). Sequence data for the remaining studies was not included since it was not publicly available, the corresponding authors did not provide it following request [27, 28], or the data was published without information regarding disease status of the samples[26].

**Table 1:** Characteristics of study cohorts included in the analysis.

Study Design	Time-point of bio-specimen collection	DNA Extraction	PCR Primers	Target region	Sequence Platform	Samples	Data shared
**Marchesi | Tjalsma, 2011**: Tumor:tumor-adjacent biopsy	Samples collected at surgical resection	AllPrep DNA/RNA kit, Qiagen	27f/1492r, L1401r/968f-GC	V1_V3	454 FLX Titanium	CRC-6, Ctrl-6, Total-12	✔
**Kostic | Meyerson, 2012**: Tumor:Tumor-adjacent biopsy	Samples collected from University Hospital in Barcelona and Genomics Collaborative inc, exact time-point not mentioned	Bass et al/Not mentioned	375F, 926R	V3_V5	454 FLX Titatnium	CRC-95, Ctrl-95, Total-190	✔
**Chen | Xiang, 2012**: Tumor:tumor-adjacent biopsy, paired fecal samples from a subset of CRC cases	At the time of surgery, included in the study if patients had not received any prior treatment for cancer and had not taken antibiotics for at least a month prior to sample collection	QIAamp DNA Kit	27F, 533R	V1_V3	454 FLX Titanium	CRC-27, Ctrl-27, Total-54	✔
**Geng | Zhang, 2013**: Tumor:tumor-adjacent biopsy	At colonoscopy	QIAamp DNA Kit	27F, 338R	V1_V2	454 FLX	CRC-8, Ctrl-8, Total-16	✔
**Weir | Ryan, 2013:** Tumor:tumor-adjacent biopsy, paired fecal samples from a subset of CRC cases	Prior to colonic resection surgery, no antibiotics for two months	MoBio Powersoil	515F, 806R	V4	454-FLX	CRC-7, Ctrl-7, Total-14	✔
**Zeller | Bork, 2014**: Tumor:Tumor-adjacent biopsy	Prior to bowel prep for colonoscopy and resection surgery	G'NOME DNA	515F, 806R	V4	Illumina-MiSeq	CRC-48, Ctrl-48, Total-96	✔
**Nakatsu | Sung, 2015:** Tumor:Tumor-adjacent biopsy	At screening colonoscopy, excluded patients with a history of CRC, IBS, IBD	QIAamp DNA Kit	27F-800R	V1_V4	454 FLX+ Titanium	CRC-102, Ctrl-86 Total-188	✔
**Burns | Blekhman, 2015**: Tumor:Tumor-adjacent biopsy	Patient samples obtained from a consortium, time of sample collection not mentioned	Qiazol lysis solution followed by sonication in an ultrasonic heat bath	787-803F, 1046-1064R	V5_V6	Illumina MiSeq	CRC-44, Ctrl-44, Total-88	✔
**Mira-Pascual | Collado, 2015**: Paired fecal and biopsy samples from the same CRC case	During colonoscopy	Macherey–Nagel, Germany	27F, 533R	V1-V3	454-FLX	CRC-9, Ctrl-5, Total-14	✔
**Dejea | Sears, 2016**: Tumor:Tumor-adjacent biopsy	Samples collected at the time of surgery, patients with a previous history of CRC or who received treatment for cancer and had taken antibiotics in the last three months prior to surgery were excluded	Following pressure lysis, DNA was extracted using the QIAamp DNA extraction kit	375F, 926R	V3_V5	454 FLX Titatnium	CRC-45, Ctrl-25, Total-70	✔
**Flemer | O'Toole, 2016**: Tumor:Tumor-adjacent biopsy, paired fecal samples from a subset of CRC cases	Samples collected at colonic resection (CRC and CRA) and at screening colonoscopy for controls, excluded if having previous history of IBS, IBD and antibiotic use in one month prior to the surgery	AllPrep DNA/RNA kit, Qiagen	Custom	V3_V4	Illumina MiSeq	CRC-59, Ctrl-56, Total-115	✔
**McCoy | Keku, 2013:** Tumor:Tumor-adjacent biopsy	UNC Tissue Procurement Facility. Exact time not mentioned	Qiagen DNeasy Blood and Tissue Kit	27F, 338R	V1_V3	454 FLX Titanium	CRC-10, Ctrl-9, Total-19	**X**
**Sanapareddy | Keku, 2014**: Tumor:Tumor-adjacent biopsy	At screening colonoscopy, excluded patients with previous CRC, CRA, IBD, sigmoidoscopy and FAP	Qiagen DNA isolation kit	A-8FM, B-357R	V1_V2	454 FLX Titanium	Ad-33, CRC-0, Ctrl-38, Total-71	**X**
**Gao | Qin, 2015:** Tumor:Tumor-adjacent biopsy	During resection surgery, excluded cases with previous chemotherapy and antibiotic use	MoBio Powersoil DNA extraction kits	515F, 806R	V3	454 FLX	CRC– 51	**X**

DNA: Deoxyribose Nucleic Acid, PCR–Polymerase Chain Reaction, V- Variable Region in 16S rRNA gene, in PCR primers, F- Forward, R-Reverse, Ad–Adenoma, CRC–Colorectal Cancer, Ctrl–Control, IBS- Irritable Bowel Syndrome, IBD- Inflammatory Bowel Disease, FAP–Familial Adenomatous Polyposis

All raw sequence data was analyzed using QIIME 1.8.0 [29]. Depending on the format of files available from SRA, files were converted to either sff or fastq format. Corresponding fna/qual and fastq files were demultiplexed with per-sample mapping files (including barcodes), where required, and forward/reverse primers in all other cases [6, 17, 19, 22]. Minimum and maximum length for quality filtering for the 454 study cohorts varied according to the 16S rRNA gene variable region sequenced in the study and were set to 200 and 1000 bp, respectively, for Chen et al., Weir et al., Kostic et al., and 200 and 600 bp for Marchesi et al. and Sears et al. This was achieved using split_libraries.py and set to default for the fastq files using the split_libraries_fastq.py command (*i*.*e*., we truncated reads immediately after runs of more than one consecutive low-quality base calls (q < 20) and excluded reads with < 0.75 of the original read length after truncation). Default parameters of the pick_closed_reference_otus.py command were used to create operational taxonomic unit (OTU) tables and assign taxonomy. Briefly, OTUs were clustered using UCLUST 1.2.22q [30] with the pick_reverse_strand_enabled flag set to TRUE against a reference database, Greengenes 13_8 (Table 2) [31]. In some instances, technical replicates (*i*.*e*., two samples per study participant from the same tumor or adjacent unaffected area) were available. When this occurred, we processed all the samples through the closed reference OTU picking pipeline and retained the sample yielding the greater number of sequences. In one study [6], the authors collected biopsy samples from 2 to 5 cm and 10 to 15 cm away from the CRC tissue samples. In order to maintain consistent sample definitions, these were considered ‘tumor biopsy-adjacent’ samples and were paired with their matched CRC biopsy counterparts. Samples comprised of fewer than 100 sequences were excluded from further analysis. One study [26] was excluded from downstream analysis due to consistently low sequence yields across multiple samples.

**Table 2:** Study-wise sequence analysis statistics.

Study Abbreviation	Source of data	Count of raw sequence reads	QC reads	Fraction of QC reads assigned to OTUs	Fraction of raw reads assigned to OTUs	Avg reads ± SD/biospecimen
Marchesi_V13_454_2011	Shared by author	5 79 736	33.90%	77.60%	26.30%	12748.8 ± 72743.1
Kostic_V35_454_2012	NCBI SRA	10 71 252	58.20%	60.60%	35.30%	1 972.2 ± 1 675.8
Chen_V13_454_2012	NCBI SRA	4 74 186	72.40%	82.40%	59.70%	3538.8 ± 1041.5
Geng_V12_454	NCBI SRA	65 491	3.60%	78.80%	2.80%	116.4 ± 48.8
Weir_V4_454_2013	Shared by author	96 583	40.70%	23.40%	9.50%	614.1 ± 559.2
Zeller_V4_MiSeq_2014	EBI ENA	1 46 28 665	97.50%	93.40%	91.10%	143360.2±73962.9
Nakatsu_V14_454_2015	NCBI SRA	39 45 849	74.10%	40.40%	29.90%	4297.9 ± 2737.2
Burns_V56_MiSeq_2015	NCBI SRA	1 40 31 598	81.10%	10.30%	8.40%	13 388.1 ± 14 687.4
Pascual_V13_454_2015	MG-RAST	1 50 801	41.80%	95.40%	39.90%	1 627.3 ± 1658.8
Sears_V35_454_2016	NCBI SRA	8 14 332	55.50%	89.40%	49.70%	5 620.5 ± 5 836.0
Flemer_V34_MiSeq_2016	NCBI SRA	51 34 339	62.20%	89.80%	55.80%	12 259.1 ± 5 960.8

Abbreviations: QC: Quality Controlled, OTU: Operational Taxonomic Unit, Avg: Average, NCBI: National Center for Biotechnology Information, SRA: Sequence Read Archive, EBI: European Bioinformatics Institute, ENA: European Nucleotide Archive, SD: Standard Deviation

### Statistical analysis

All statistical analyses were performed using R software (version 3.2.1). Samples from patients having received chemotherapy or radiotherapy were excluded from analysis and OTUs occurring in < 5% of all samples were excluded. Principle co-ordinates analysis plots of an OTU- based Bray-Curtis dissimilarity matrix were generated for the fecal-carcinoma paired samples and biopsy-control paired samples [32]. A unique aspect of the experimental 'paired' design was pairing phenotypically healthy tumor-adjacent tissue or fecal sample with tumor biopsy specimens from the *same* CRC case. Procustes analyses were performed using the ade4::procuste function [33], which uses uniform scaling (expansion or contraction) and rotation to minimize squared differences between CRC tumor and tumor-adjacent biopsy or CRC biopsies and fecal sample ordinations. A permutation-based test using vegan::protest was used to test the null hypothesis that the degree of congruence was greater than random between sample pairs [33, 34].

To minimize the impact of experimental biases stemming from uneven sequencing depths across studies and high dimensionality of closed reference OTUs, the OTU table was filtered to retain high abundance taxa, which were then agglomerated to the genus level. Specifically, taxa with relative abundances greater than the mean of the distribution for each taxon across all samples were retained for further analysis. Relative abundances of major phyla were compared using a k-sample permutation based test for each of the following sample types: tumor biopsy, tumor-adjacent biopsy, and paired fecal:biopsy from the same CRC host.

A per-study DESeq2 analysis was used to evaluate the differential abundance of genera in (a) the CRC tumor:tumor-adjacent biopsy comparison, and (b) tumor biopsy:fecal samples comparison, adjusting for paired design (*i*.*e*., samples collected from the same host) [35, 36]. Log_2_fold changes and standard errors obtained from the DESeq2 analysis were used as effect size estimates and corresponding sampling variances, respectively. A random effects (RE) model controlling for study as the random effect was generated using the metafor package [37]. Genera present in ≥ four of the tumor:tumor-adjacent biopsy comparisons or ≥ three of the fecal:biopsy comparisons (*i*.*e*., 50% of studies) were retained for random effects analysis. FDR correction was applied to each of the RE model p-values to account for multiple testing across all of the models.

Using caret [38], a random forest (RF) classifier was used to assess the degree to which microbial signatures were capable of distinguishing tumor from tumor-adjacent or biopsy from matched fecal sample types. Combined relative abundance-transformed genus-level counts across all studies were used as an input for RF analysis. The number of predictor features randomly sampled for splitting at each node in the decision tree, commonly known as *mtry*, was tuned as (0.5, 1, 1.5, 1.75, 2, 2.5, 3.0)*(square root of total number of microbial predictors). Models were internally cross-validated ten-fold times with five repeats to avoid over-fitting. The tuning area under receiver operating characteristic (AUROC) curve presenting the largest value was used to select the optimal model and was plotted using the pROC package (Robin et al. 2011). Differences in AUROC were analyzed statistically with DeLong’s test [39].

To identify potential functional differences between tumor:tumor-adjacent biopsy and paired tumor:fecal samples, metagenomic content was inferred from 16S rRNA gene sequence data using PiCRUST 1.0 [40] and version 54 of the KEGG [41] database. This version includes approximately 7,000 annotated bacterial reference genomes. Copy numbers for the 16SrRNA gene were normalized by normalize_by_copy_number.py followed by the predict_metagenome.py function [40]. The FishTaco pipeline was utilized to score the marginal contribution of taxa associated with the changes in predicted metagenomic functions using Shapley value analysis which works out the relative importance of predictor variables in linear regression [42]. A taxa-based functional profile of each sample was first constructed as a linear combination of the community members’ genomic content, weighted by their abundances. A permutation-based approach was then employed. This compared the functional shifts observed in the taxa-based functional profiles when a taxon’s relative abundance was shuffled across samples to the shifts observed when this taxon’s abundance was not shuffled. This analysis helped in determining whether differences in inferred metagenomic function were due (in large part) to single organisms (e.g., Fusobacterium) or multiple organisms (i.e., the sum of the parts being greater than the effect of single organisms alone).

## Results

**Fig 1:**
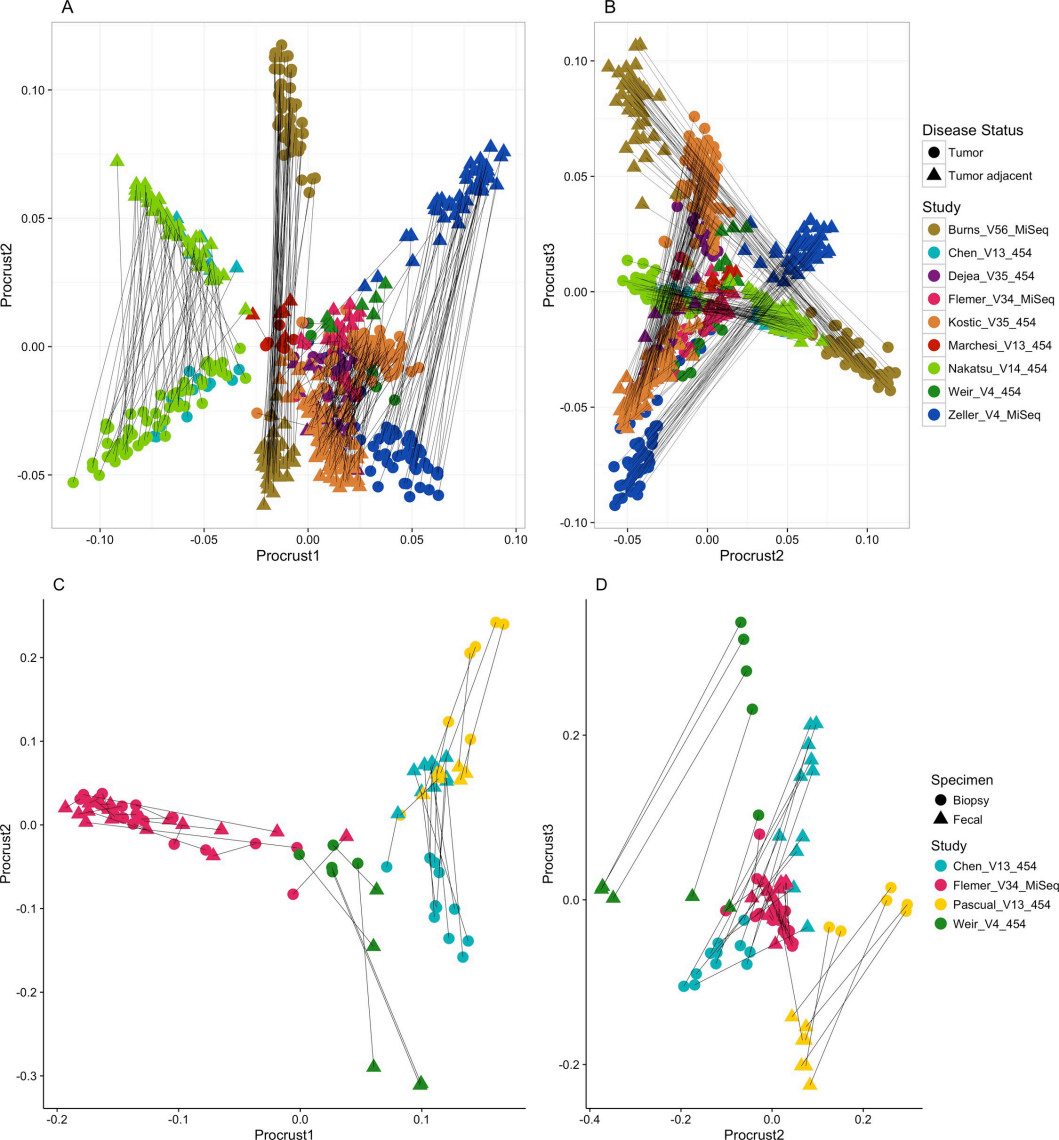
Graphical comparison of CRC tumor:tumor-adjacent tissue (1A and 1B) and paired fecal vs. tumor biopsy (1C and 1D) microbiome configurations using Procustes analysis. In Fig 1, the Procustes analysis showed a moderate [in magnitude] but statistically significant difference between both the paired tumor and tumor-adjacent biopsy (**Fig 1A and 1B**) microbiome (m^2^ = 0.68, p < 0.001) as well as paired fecal and CRC tumor tissue samples (**Fig 1C and 1D**); m^2^ = 0.65, p < 0.001) from the same case of CRC. Lines connect paired samples. Shapes indicate sample phenotype; colors indicate study cohort.

Microbial profiles were analyzed from a total of ten colorectal cancer associated studies, comprising 588 matched tumor and tumor-adjacent specimens (n = 294 pairs from nine studies) and 84 matched fecal and tumor biopsy specimens (n = 42 pairs from four studies; Tables 1 and 2). Principal coordinate analysis (PCoA) of paired tumor:tumor-adjacent samples revealed that these communities clustered primarily by study, then by platform and gene target. Although separation between these microbial communities was discernable, it was not completely distinct (S1 Fig). Tumor biopsy:fecal pairs from the same CRC case showed a compositional change in taxon abundances, especially in the investigations conducted by Chen et al. (Chen_V13_454) and Mira-Pascual et al. (Pascual_V13_454); (Panel A in S2 Fig). This difference was even more apparent when the PC3 axis was plotted against PC4 (Panel B in S2 Fig). Procustes rotation revealed a moderate degree of similarity in most paired tumor: tumor-adjacent samples, while even greater similarity was observed in the studies conducted by Marchesi et al. (Marchesi_V13_454), Dejea et al. (Dejea_V35_454), Weir et al. (Weir_V4_454), and Kostic et al (Kostic_V35_454);(Fig 1A and 1B). The overall correlation was 0.68 for axis 1 vs 2 (sum of squared deviations m^2^ = 0.53) and 0.85 for axis 2 vs 3 (m^2^ = 0.27 [values for m^2^ range from 0 (matrices are highly similar) to 1 (matrices are dissimilar)]), with p = 0.001, rejecting the null hypothesis that the degree of congruence between the two Procustes matrices is no greater than random (Fig 1A and 1B). The same Procustes graphical super-imposition showed a separation between the matched CRC tumor tissue and fecal samples (m^2^ = 0.57 for axis 1 vs 2 and 0.25 for axis 2 vs 3, permutation-based p-value = 0.001; Fig 1C and 1D).

**Fig 2:**
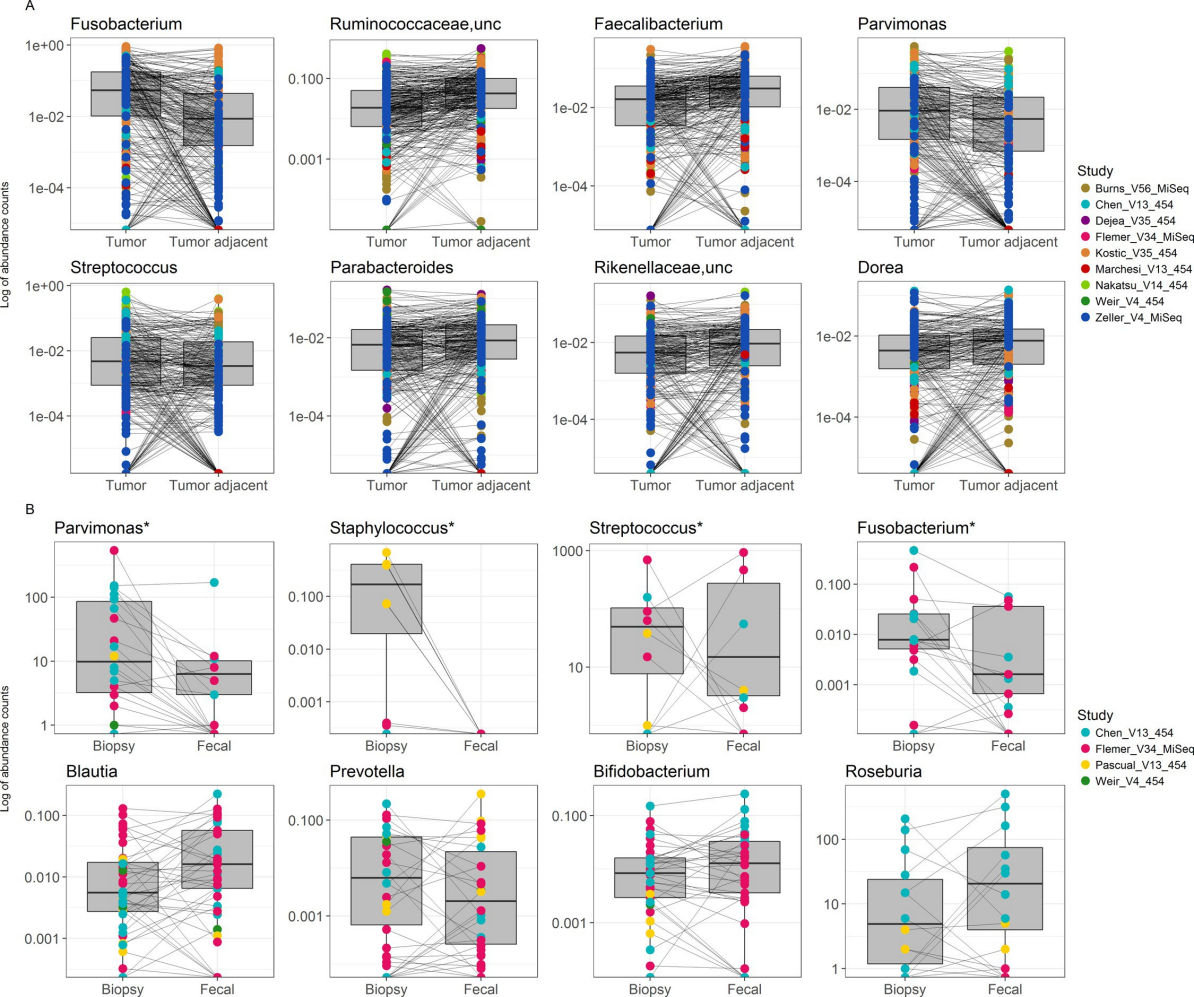
Pairwise differences in tumor vs. adjacent tissue and fecal vs.tumor biopsy samples. Boxplots indicate the distribution of the relative abundances of various taxa and corresponding lines connect paired samples, depicting the direction of change in relative abundance of statistically significantly different families between CRC tumor biopsy samples (left) and adjacent non-affected tissue microbiome (**Fig 2A**, n = 294 pairs, 588 samples) or fecal sample (**Fig 2B**, n = 42 pairs, n = 84 samples) for the various studies (colors) * indicates mean relative abundance was statistically significantly different between the genera by paired Wilcoxon signed rank test and p<0.05 after FDR adjustment. All biopsy-based taxa presented in Fig 2A were statistically significantly different between tumor and tumor biopsy samples by above mentioned test.

Phylum-level differences revealed that CRC tumor biopsy specimens harbored greater abundances of Fusobacteria and Actinobacteria, while their paired adjacent tissue counterparts harbored an elevated abundance of Firmicutes. Compared to their tumor biopsy counterparts, fecal samples harbored greater abundances of Verrucomicrobia and Euryarcheota and fewer Proteobacteria (S3 Fig). In a pair-by-pair comparison of the most abundant annotated genera, CRC tumor samples exhibited greater mean abundances of *Fusobacterium* and *Parvimonas* while tumor-adjacent samples presented greater mean abundances of Ruminococcaceae, *Faecalibacterium* and *Parabacteroides* among others (Fig 2A). In the matched comparison, fecal samples yielded greater mean abundances of *Roseburia*, *Blautia*, and *Bifidobacterium* while biopsy samples harbored greater mean abundances of *Fusobacterium*, *Streptococcus*, *Prevotella*, and *Staphylococcus* (Fig 2B). Within paired samples, there was considerable intra- and inter-study heterogeneity with respect to the magnitude and direction (elevated versus attenuated in tumor biopsy) of taxonomic changes. That said, a small number of taxa, *e*.*g*., *Fusobacterium*, *Parvimonas*, and *Streptococcus* were consistently detected in greater abundance in tumor-associated samples, compared to both adjacent tissues and feces.

**Fig 3:**
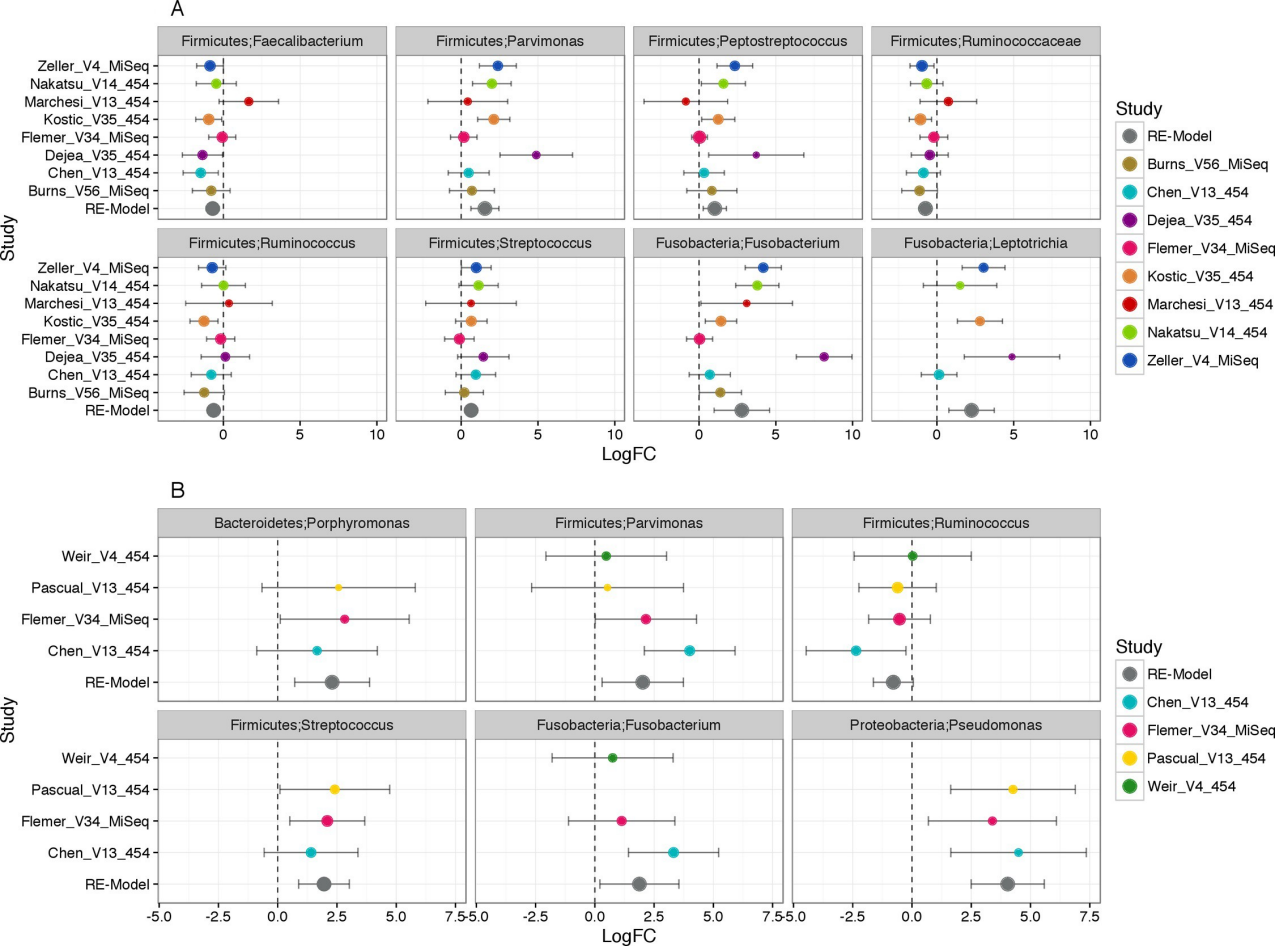
Forest plot of taxa whose abundance is consistently differential. Plots depict per study and adjusted (REM model) log-fold change across all studies for taxa that were differentially abundant in >50% of available studies i.e ≥ five of the eight studies with paired CRC biopsy samples (shift to right indicates taxa elevated in tumor; shift to left indicates taxa elevated in tumor adjacent biopsy) in **Fig 3A** and ≥ three studies of the total four for the paired CRC fecal and biopsy samples studies (i.e., for both Fig 3A and 3B) (to the right indicates taxa elevated in tumor biopsies and to the left indicates taxa elevated in fecal CRC case) in **Fig 3B**. Individual log fold changes and FDR p-values for paired biopsy and paired fecal comparisons are provided in S2 and S3 Tables, respectively. Error bars denote 95% confidence intervals, size of point indicates the precision of the point estimate for individual studies [1/ (95% CI Upper Bound– 95% CI lower bound)]. REM-model point size is fixed. Blank values for a particular study indicate that DESeq2 did not determine that taxa to be differentially abundant in that particular study cohort.

To identify robust, genus-specific associations across all studies, we performed differential abundance testing which accounted for the paired study design by assigning a ‘pair factor id’ to matched samples. Results from this per-study DESEq2 evaluation for 294 tumor:tumor adjacent biopsy pairs were compared across the nine studies with a random effects model. Of the 80 genera analyzed, 41 were identified as being differentially abundant in 5 or more studies (i.e., >50% of studies analyzed), and 5 of these genera remained significant after FDR adjustment (p ≤ 0.1). Consistently observed were the increased abundances of *Fusobacterium* spp. (8/8 studies, adjusted REM model Log_2_fold change: 2.6, 95% CI: (0.9, 4.5), p = 0.002, FDR p = 0.02), *Leptotrichia* (5/8 studies, adjusted REM model Log_2_fold change: 1.4, 95% CI: (0.7, 3.7), p = 0.002, FDR p = 0.02), and *Parvimonas* (8/8 studies, adjusted REM model Log_2_fold change: 1.5, 95% CI: (0.6, 2.5), p < 0.001, FDR p = 0.001), along with *Peptostreptococcus* and *Streptococcus*, in tumor biopsy tissues relative to tumor-adjacent tissues. In contrast, an unclassified genus in the family Ruminococcaceae (8/8 studies, adjusted REM model Log_2_fold change: -0.7, 95% CI: (-1.1, -0.4), p = 1.9*10^−5^, FDR p = 0.001) and species of *Faecalibacterium* (8/8 studies, adjusted REM model Log_2_fold change: -0.7, 95% CI: (-1.1, -0.3), p = 0.001, FDR p = 0.02) were significantly more abundant in adjacent tissues than in tumor-associated specimens (Fig 3A and S2 Table).

In evaluating fecal and biopsy samples from the same CRC case, a total of 42 pairs (n = 84 samples) from four distinct studies were considered. Of the 73 genera detected among these samples, 38 were differentially abundant in at least three of the four cohorts (i.e., >50% of studies analyzed), and three genera were significantly differentially abundant by the REM. These included the observed increase in abundance of *Pseudomonas* (3 of 4 studies, adjusted REM model Log_2_fold change: 4.0, 95% CI: (2.5, 5.5), p = 2.8*10^−7^, FDR p = 1.1*10^−5^), *Streptococcus* (3 of 4 studies, adjusted REM Log_2_fold change: 1.9, 95% CI: (0.8, 3.0), p < 0.001, FDR p = 0.006), and *Porphyrmonas* (adjusted REM Log_2_fold change: 2.3, 95% CI: (0.7, 3.8), p = 0.004, FDR p = 0.05) in tumor-associated specimens relative to fecal samples. Although *Fusobacterium* and *Parvimonas* exhibited high REM adjusted Log_2_fold change values (1.8 in 3 of 4 studies and 2.0 in 4 of 4 studies, respectively), these did not retain statistical significance after FDR correction (Fig 3B and S3 Table). Per the RE model, four taxa were common across the paired biopsy and biopsy:fecal comparisons: species of *Parvimonas*, *Porphyrmonas*, *Phascolarctobacterium*, and *Lachnobacterium*.

**Fig 4:**
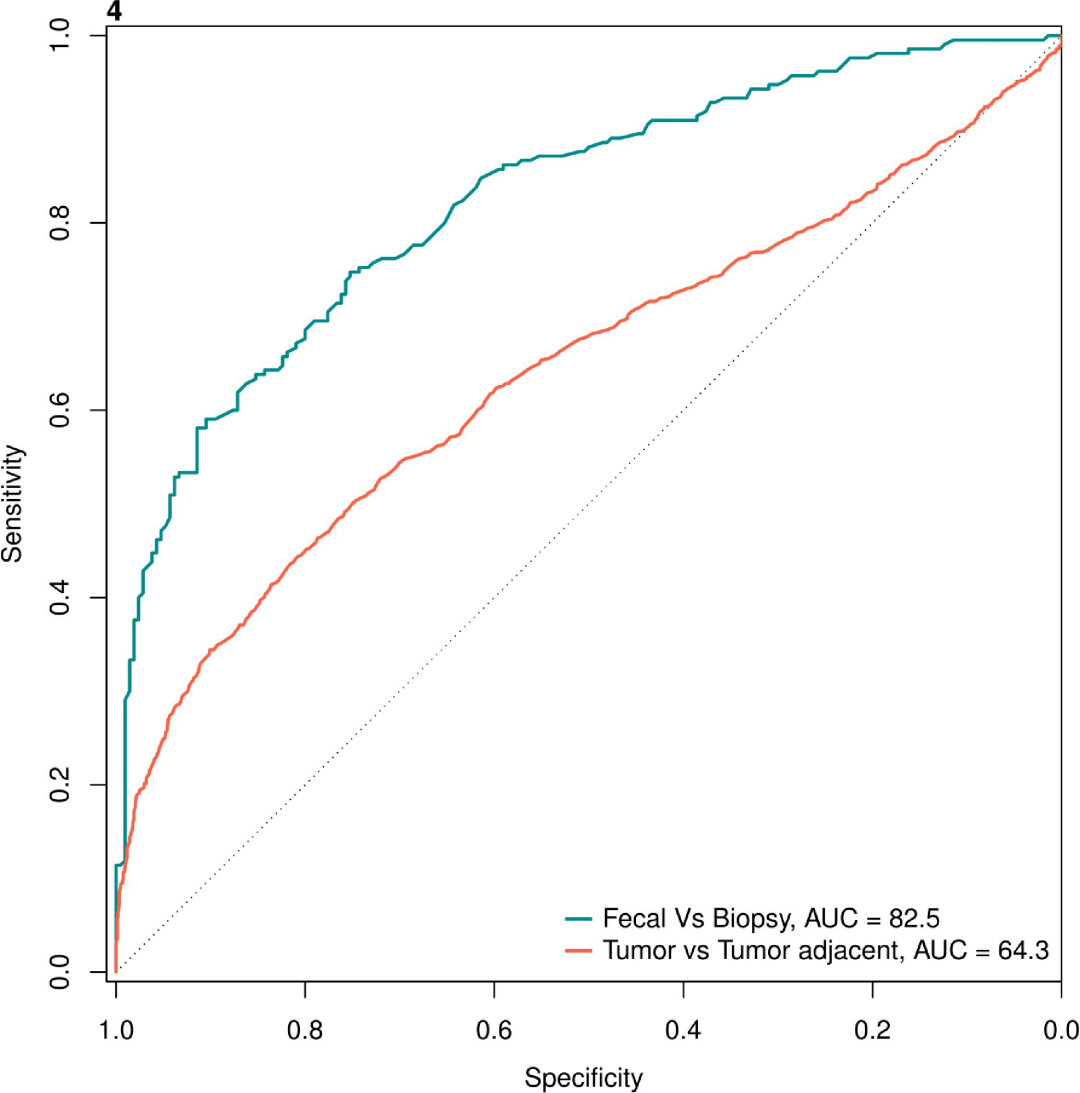
Microbial taxa-based models for distinguishing CRC tumor-associated microbiome from tumor adjacent tissue-associated and fecal-associated specimens. The tumor biopsy vs. fecal classifier [area under curve (AUC) = 82.5] was better able to distinguish CRC fecal samples from tumor tissue samples than tumor vs. tumor adjacent biopsy classifier (AUC = 64.3). Again, given the compositional overlap between these niches, these classifiers relied on differentially abundant features rather than niche-specific distribution.

We evaluated the similarity (and dissimilarity) of taxa in biopsies and fecal samples. Of the 35 non-zero abundance genera present in both, 6 were unique to biopsies, 21 were present in biopsies as well as fecal samples while fecal samples had an additional 8 unique taxa (S4 Table). A random forest classifier to distinguish mucosal and fecal associated taxa performed with reasonable accuracy. With an area under the ROC curve of 82.5% (Fig 4), the taxa contributing to differentiation between the two sample types were members of the phylum Proteobacteria (Panel B in S4 Fig). It should be noted that the fecal-biopsy classifier was based on the relative abundances of microbial features rather than their simple presence or absence. We found many overlapping taxa between these ecological niches, and the RF model demonstrates that although the distribution of these taxa is shared, their richness or density vary based upon niche. The random forest model for classifying paired tumor biopsy samples and tumor-adjacent tissues exhibited an area under the ROC curve of 64.3% (Fig 4), suggesting that tumor-adjacent tissues harbor microbial communities that are more difficult to distinguish from, and thus more similar to, tumor-associated communities than tumor versus stool-associated communities. The more discriminatory taxa for the paired biopsy samples included those within the genera *Fusobacterium* and *Faecalibacterium *(Panel A in S4 Fig).

**Fig 5:**
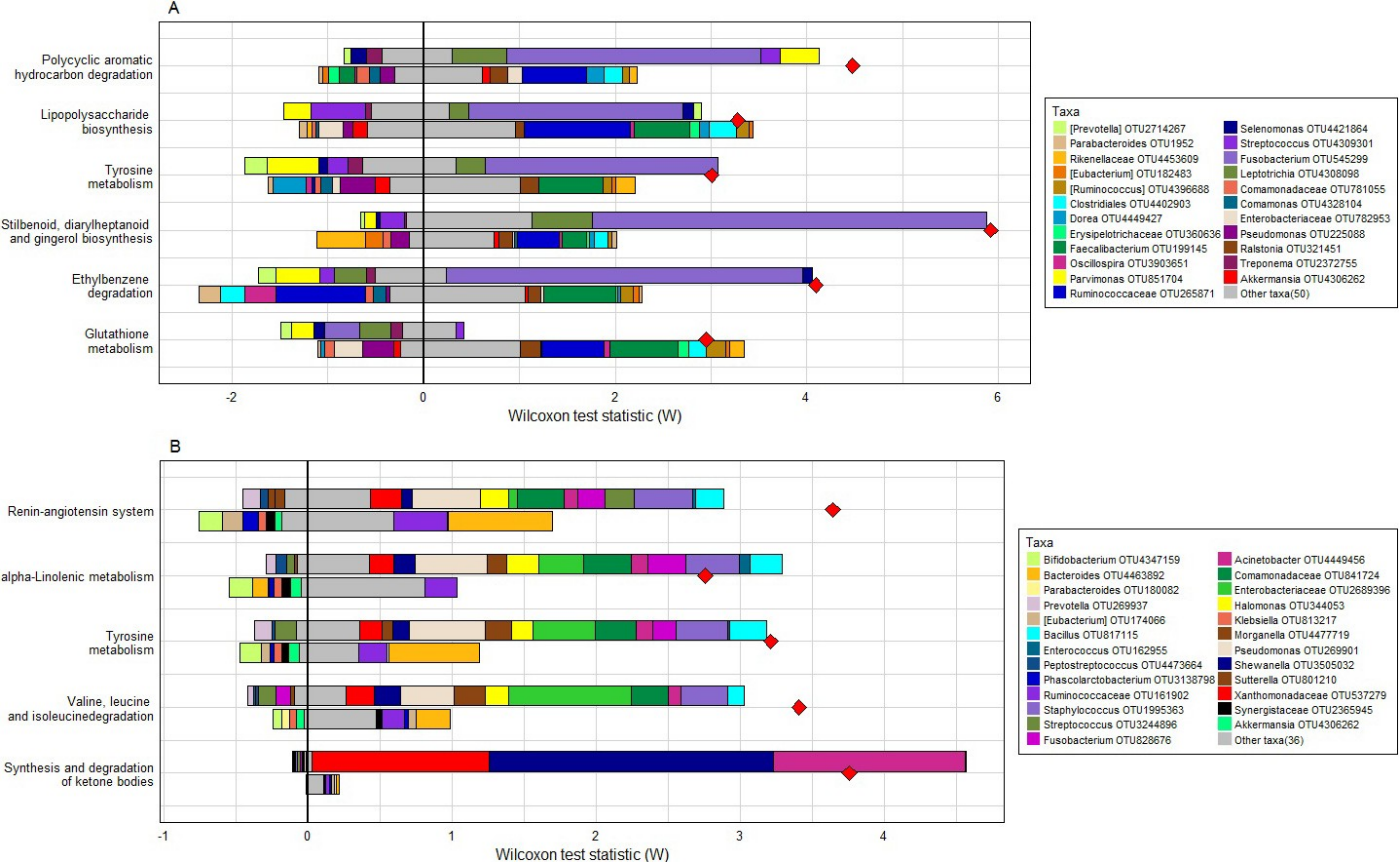
Comparative analysis of imputed functional groups contributed by various bacterial taxa. For each pathway presented, the top left bar shows the tumor biopsy-associated taxa that attenuate the functional shift, the top right bar shows the tumor biopsy-associated taxa that are associated with an increase in the functional shift magnitude, and the bottom bars are referring to **Fig 5A:** tumor-adjacent taxa or **Fig 5B:** fecal-associated taxa. OTUs mentioned in the legend are OTUs classified to genus level. Red diamond markers indicate the cumulative metagenome-based shift in Wilcoxon score. In Fig 5A, tumor (top bar): tumor-adjacent biopsy (bottom bar) samples, *Fusobacterium* and *Leptotrichia* are tumor biopsy associated and related with increased function. *Parvimonas*, is also tumor biopsy associated but related with attenuated functional shifts for most pathways. On the other hand, in Fig 5B, in tumor biopsy (top bar) and fecal samples (bottom bar) obtained from the same CRC patient, several different Proteobacteria (e.g., Xanthomonadaceae, Comamonadaceae, Enterobacteriaceae, *Halomonas*, and *Morganella*) were associated with tumor biopsy and enrichment of the functional pathways.

The final aim of this study was to determine which functional differences may be present in tumor-associated communities and the degree to which these differences may be driven by the primary taxonomic perturbations we identified or were the result of subtle shifts among multiple taxa. The single-taxon filter in FishTaco was used to identify 14 differentially abundant KEGG pathways. Of these, six statistically significant pathways remained after being further evaluated in the multi-taxa mode (accounting for taxa co-variation) and subjected to multiple comparison adjustment. The relative abundances of pathways for tyrosine metabolism, glutathione metabolism, lipopolysaccharide (LPS) biosynthesis, polycylic aromatic hydrocarbon degradation, ethylbenzene degradation, and stillbenoid, diarylheptanoid and gingerol biosynthesis differed significantly between tumor and tumor-adjacent tissue samples. Species of *Fusobacterium* and *Leptotrichia* were the primary CRC case-associated taxa associated with enrichment of tyrosine metabolism, LPS biosynthesis, and polycyclic aromatic hydrocarbon degradation (Panel A in Fig 5).

In a paired tumor biopsy:fecal comparison, single-taxon permutation analyses identified 13 differentially abundant KEGG pathways that, when subject to multi-taxa analysis coupled with Shapley orderings, yielded a total of six statistically significant functional pathways. These included synthesis and degradation of ketone bodies, which were largely impacted by differing abundances of Xanthomonadaceae, *Shewanella*, and *Acinetobacter (*all belonging to Phylum Proteobacteria). *Pseudomonas*, members of the families Comamondaceae and Enterobacteriaceae, and *Staphylococcus* contributed marginally to valine, leucine, and isoleucine degradation, tyrosine metabolism, alpha-Linolenic metabolism, and the renin-angiotensin system (Fig 5B).

## Discussion

In this pooled analysis, we sought to identify bacterial taxa whose relative abundance consistently altered in multiple cohorts evaluating CRC tumor biopsies. Efforts were made to determine how these samples differed from physically adjacent non-tumorous tissue, and the extent to which they were represented in fecal specimens, which can be used non-invasively for colorectal cancer screening and diagnosis. Statistically adjusting for the paired design with tumor and tumor-adjacent biopsy/fecal samples from the same individual and controlling for inherent genetic and environmental differences that may occur in different hosts, we surveyed changes in microbial population composition and potential metabolic function. A limited number of taxa elaborated below were confirmed by multiple 16S rRNA gene sequencing (mucosal or fecal) based datasets while many findings identified by individual studies were not.

An elevated prevalence and abundance of *Fusobacterium* was observed, which corroborated previous reports. *Fusobacterium* was frequently accompanied by an increased abundance of *Leptotrichia*, members of the same bacterial family. Certain species of these genera are oral commensals that can elicit pathogenesis outside of the oral niche. Mechanistic studies have established that *Fusobacterium nucleatum*’s FadA adhesin binds to cell-cell adhesion molecule E-cadherin which activates β-catenin signaling and promotes CRC cell proliferation[9]. *F*. *nucleatum* also acts as persistent anchor of biofilms in the cancer tissue and subsequent E-cadherin loss activates Wnt signaling and IL-6 driven Stat3 activation. While some studies have established a higher presence of *F*. *nucleatum* in adenomas as compared to healthy tissue, some have not found a significant difference in *Fusobacterium* levels in stool samples of adenoma patients as compared to healthy participants [28, 43]. Thus, it is possible that *Fusobacterium* is localized in the mucosal tissue during pre-cancerous polyp formation and becomes potentially more abundant and detectable in fecal samples as colorectal adenoma progresses to adenocarcinoma thus rendering further support to the previously established on-tumor off-tumor community concept [13].

Species of *Parvimonas* were consistently displayed elevated abundance in tumor biopsies. *P*. *micra*, the only species described in the genus [44], is known to cause bacteremia, abdominal abscesses, endocarditis, and other infections [45]. Recent studies have implicated *Parvimonas* in CRC disease [4, 6, 14, 18], and we confirm this association through random effects modeling. In some cases, nucleic acid sequences belonging to members of this genus were detected in a large proportion of CRC biopsy samples (Fig 3A and 3B), even though they were not explicitly reported by the original authors of these investigations [2, 17].

*Streptococcus* was also significantly differentially abundant in all tumor:tumor-adjacent tissue and most tumor biopsy:fecal comparison studies. A recent study by Kumar et al. demonstrated that mice inoculated with *S*. *gallolyticus* subsp. *gallolyticus* exhibited significantly more tumors and an elevated grade of dysplasia. This effect was abolished by knocking down β-catenin, hinting at an effect measure modifier role for the pathogen [46].

Nucleic acid signatures of bacteria belonging to the genera *Parvimonas*, *Fusobacterium* and *Streptococcus*, taxa previously described as having diagnostic potential in stool [4, 20, 21], were consistently detected in tumor tissue (*i*.*e*., at the disease interface). These organisms occurred in greater abundances in tumor biopsy samples than they did in stool, but their consistent detection in stool suggests that they may non-invasively reflect, in part, the biology of disease microenvironment. Microbiome-based diagnostics promise great potential for detecting CRC however will require rigorous validation in the context of the stage of the disease, co-morbid conditions and generalizability to the population[47].

While this manuscript was being prepared, the results of a similar investigation were published [21].The authors aggregated findings from different 16S rRNA gene sequencing based cohorts. Taxa identified in the fecal portion of their study overlapped with those previously reported by our group [4], and the AUC of their microbial tumor tissue classifier was similar to that reported here. Although both their study and ours lend support to one another with respect to the potential to identify CRC-associated microbial markers in stool and tumor tissues, a key difference between our studies is that Sze et al. did not observe consistently elevated abundances of *Fusobacterium*, *Parvimonas*, or *Streptococcus* associated with tumor tissue samples. This could be the result of having leveraged different collections of cohorts, different pipelines for analyzing 16S rRNA gene sequence data, and/or different statistical models and effect measure estimates (i.e., Log2ratios in our study compared to Odds Ratios in their study).

The abundances of several OTUs belonging to *Ruminococcus* and *Faecalibacterium* were consistently elevated in tumor-adjacent tissues and fecal CRC samples, compared to the tumorous counterparts. This reduced abundance of Lachnospiraceae and Ruminococcaceae OTUs in CRC have been previously reported in CRC microbiome studies [1, 6, 48]. Microbial signatures capable of differentiating adjacent mucosa from tumorigenic tissue could prove extremely valuable in detecting stages of carcinogenesis and potentially identifying the tipping point in malignant transformation. Some studies have reported a partially overlapping spectrum of microbial taxa in these closely located sites [14, 18], which may be attributed to diffusion associated with the tumor site and/or leakage from angiogenic channels capable of transporting microbes and or microbial remnants to regions adjoining the tumor tissue.

Metagenomic prediction suggested that *Fusobacterium*, *Leptotrichia*, and *Streptococcus* appear to be largely responsible for case-associated enrichment of tyrosine metabolism in both tumor:tumor-adjacent and tumor:fecal comparisons. Tyrosine kinase mediates angiogenesis, the process by which cancer cells receive nutrients through blood circulation [49], as well as the acute IL-8 induced inflammatory response driven by *B*. *fragilis* [50]. It seems plausible, then, to consider tyrosine metabolism as a functional target for attenuating cancer pathogenesis. Being gram-negative, *Fusobacterium*, *Leptotrichia*, and *B*. *fragilis* have dense lipopolysaccharide (LPS) outer membranes and high densities of these taxa detected in tumor biopsy tissue supports the notion that LPS biosynthesis can be considered to be a case-associated pathway. This biochemical is pro-inflammatory, affects lumen-epithelial barrier function by increasing intestinal tight junction permeability via localization of TLR-4 and CD14 proteins, and genes associated with its production have been described as enriched in fecal metagenomes of CRC patients [20, 51].

Other predicted pathways that were differentially abundant included valine, leucine, and isoleucine degradation, the renin-angiotensin pathway (RAS), and the synthesis and degradation of ketone bodies. Branched chain amino acids are known to serve as important nutrient signals for proliferation of immune cells in the mTOR pathway, and, like lipopolysaccharides, functional genes associated with their degradation have been described as enriched in the fecal metagenomes of CRC patients [20, 52, 53]. Numerous retrospective analyses have demonstrated a reduction in colorectal cancer incidence, polyp formation, and distant metastasis in patients taking RAS inhibitors [54], and it has been suggested that a ketogenic diet aids in managing cancers as malignant cells depend on glucose as fuels and cannot metabolize fatty acids [55]. Increased abundances of members of the Enterobacteriaceae, Comamonadaceae, *Staphylococus*, and *Fusobacterium* and a decline in observed abundances of Ruminococcaceae, *Faecalibacterium*, and *Bacteroides* were underlying themes across all of the pathways evaluated. Altered abundances of these bacterial lineages may substantially contribute to the observed responses to chemotherapeutic drugs via differential ability to metabolize various xenobiotic compounds [56, 57].

Although this study was successful in unifying data and making inferences from multiple cohorts, it was, nonetheless, bound by limitations. Substantial heterogeneity existed among these samples with respect to their pre-bioinformatics and downstream sequence processing. Previous reports have demonstrated that resulting microbial community representation across studies may be influenced by DNA extraction methods [58], primer choice and the region of 16S rRNA gene sequenced, read length and sequencing platform, sequence quality, and bioinformatics pipeline [59, 60]. Although many of these factors were beyond our control, all attempts were made to minimize bias wherever possible. This included the utilization of uniform sequence processing, bioinformatics pipelines, and appropriate statistical analyses.

Additional details pertaining to clinical and demographic factors of the participants, location of the tumor in the colon, and stage and grade of tumor were not available for all of the participants. Any and all of these could be potential confounders of the disease association with the microbiome [61]. Sharing critical clinical data along with relevant microbiome sequence information will facilitate making reliable, reproducible associations. The authors urge the scientific and medical communities to take an active stance to incentivize the sharing of such data while publishing studies. This study considered a relatively low number of matched fecal and tumor tissue sample sets, and the publication of more studies addressing this particular comparison will help shed light on differences in the microbiome and their contribution to CRC pathology in these unique niches. In this study, functional pathway information was inferred and should be interpreted with caution. Metagenomic sequencing of CRC specimens will help further validate these claims, however, in the absence of viable host-depletion techniques, shotgun metagenomic sequencing of tumor-associated microbial communities results in a high degree of host-based signal. Encouragingly, data from fecal metagenomes does support a number of our functional predictions. Finally, the SS-UP pipeline validated for fecal sample analysis in our previous manuscript [4] yielded superior taxonomic resolution and predictive performance in identifying disease state. However, as this pipeline remains proprietary we were unable to use it in the current study.

Despite these shortcomings, our study constitutes a large collection of 16S rRNA gene sequence data for fecal and biopsy CRC specimens. We identified the abundances of species of *Fusobacterium*, *Parvimonas* (*P*. *micra*) and *Streptococcus*, among others, as consistently elevated, and the abundances of *Faecalibacterium* and members of the family Ruminooccaceae to be consistently depleted in both tumor biopsy and CRC case fecal samples. While few taxa were identified in both tumor and tumor adjacent biopsy, we identified case to case as well as sample to sample heterogeneity in magnitude of change of these taxa. These taxa also frequently and collectively influence common functional pathways, such as amino acid (tyrosine, valine etc) and lipid metabolism (lipopolysaccharide synthesis and ketone degradation).

Certain microorganisms have the potential to serve as infectious agents in the etiology of CRC [1]. However, unlike other malignancies, such as liver and gastric cancer where a single organism has been implicated in the disease pathology, no single organism has been observed as definitively occurring and individually sufficiently contributing to CRC development in any of the cohorts. This observation lends support to the idea that CRC may be polymicrobial in nature [62–65]. Identifying virulent microbiota and studying their differential abundance across sample sets and cohorts, the functional pathways they encode, and their expression via meta-transcriptomics offers a promising avenue for understanding the role of the microbiome in CRC and developing microbiome-based, microbiome-compatible and microbiome-aiming therapeutic interventions.

## Supporting information

S1 Fig Principal Co-ordinates Analysis (PCoA) depicting the relationship between microbial composition from different tumor:tumor-adjacent study cohorts and their phenotypes. Plot points indicate individual samples, shapes indicate disease status (circle: Tumor, triangle: Tumor adjacent) and colors indicate various studies included in the meta-analysis (Target gene and sequencing platform are also incorporated in the study acronym) (A) Communities are compared in the PC1 vs PC2 axis where cohorts cluster tightly illustrating a strong study effect followed by the gene target region sequenced and (B) PC3 vs PC4 axis which resolves the study participants further.
(TIFF)

S2 Fig Principal Co-ordinates Analysis (PCoA) depicting the relationship between microbial composition from paired tumor:fecal study cohorts and their phenotypes. Plot points indicate individual samples, shapes indicate disease status (circle: Biopsy, CRC: Colorectal cancer) and colors indicate various studies included in the meta-analysis (Target gene and sequencing platform are also incorporated in the study acronym) (A) Communities are compared in the PC1 vs PC2 axis where cohorts cluster tightly illustrating a strong study effect followed by the gene target region sequenced and (B) PC3 vs PC4 axis which resolves the study participants further.
(TIFF)

S3 Fig Distribution of major phyla across the comparison groups tumor biopsy, tumor-adjacent biopsy and fecal samples included in the study. Tumor biopsy had the highest prevalence of Fusobacteria across samples while fecal samples had a high prevalence of Firmicutes while tumor-adjacent biopsy samples demonstrated an intermediated distribution for these phyla and showed a high prevalence of Bacteroides.
(TIFF)

S4 Fig Variable importance of different random forest classifiers. This figure depicts features ranked by their importance (Top 20 features depicted, most important at top to least at bottom) in the random forest classifier built to classify CRC tumor and tumor adjacent OR fecal samples. Each row is a microbial genera. (A) The microbial tumor:tumor-adjacent classifier comprised of 588 samples (CRC tumor biopsy (n = 294) and matched CRC tumor-adjacent biopsy (n = 294)). Fusobacterium, member of Ruminococcaceae and Faecalibacterium had a highest discriminatory power in this classifier. (B) depicts the top microbial features discriminating CRC tumor biopsy samples from CRC fecal samples within the same case. Multiple members of Proteobacteria (*Pseudomonas*, *Halomonas* and *Sutterella*) were capable of distinguishing tumor biopsy samples. Some overlap is noted in the top microbial features in classifiers between S4 Fig Panels A and B. These include *Parvimonas*, Ruminococcaceae, Lachnospiraceae and *Sutterella* among others which indicates that few tumor biopsy associated markers can also be detected in the fecal content serving as a non-invasive proxy albeit at different levels of abundance.
(TIFF)

S1 Table Links to access raw data for cohorts included in the study. (DOCX)

S2 Table Differentially abundant genera in CRC tumor biopsy as compared to tumor-adjacent biopsy identified by the Random Effects Model (REM). Taxonomy follows the convention of family, genus. **Abbreviations for S2 Table:** LogFC: Log_2_Fold Change, τ^2^: The (total) amount of heterogeneity among the true effects, SE: Standard error, QE: Test statistic for the test of (residual) heterogeneity from the full model, QEp: p-value associated with QE, I^2^: For a random-effects model, I^2^ estimates (in percent) how much of the total variability in the effect size estimates (which is composed of heterogeneity plus sampling variability) can be attributed to heterogeneity among the true effects, H^2^: estimates the ratio of the total amount of variability in the effect size estimates to the amount of sampling variability, FDR: False Discovery Rate, RE:Random Effects.
(DOCX)

S3 Table Differentially abundant genera in CRC tumor biopsy as compared to fecal samples obtained from the same case identified by the random effects model (REM). Taxonomy follows the convention of family, genus. **Abbreviations for S3 Table:** LogFC: Log_2_Fold Change, τ^2^: The (total) amount of heterogeneity among the true effects, SE: Standard error, QE: Test statistic for the test of (residual) heterogeneity from the full model, QEp: p-value associated with QE, I^2^: For a random-effects model, I^2^ estimates (in percent) how much of the total variability in the effect size estimates (which is composed of heterogeneity plus sampling variability) can be attributed to heterogeneity among the true effects, H^2^: estimates the ratio of the total amount of variability in the effect size estimates to the amount of sampling variability, FDR: False Discovery Rate, RE:Random Effects.
(DOCX)

S4 Table Genera present in both fecal and mucosal samples, only in fecal samples and only in biopsy samples. (DOCX)
