# Supplementary figures and images for: Re-purposing 16S rRNA gene sequence data from within case paired tumor biopsy and tumor-adjacent biopsy or fecal samples to identify microbial markers for colorectal cancer
Source: PLoS One. 2018 Nov 9;13(11):e0207002. doi: 10.1371/journal.pone.0207002 (PMC6226189; doi:10.1371/journal.pone.0207002)

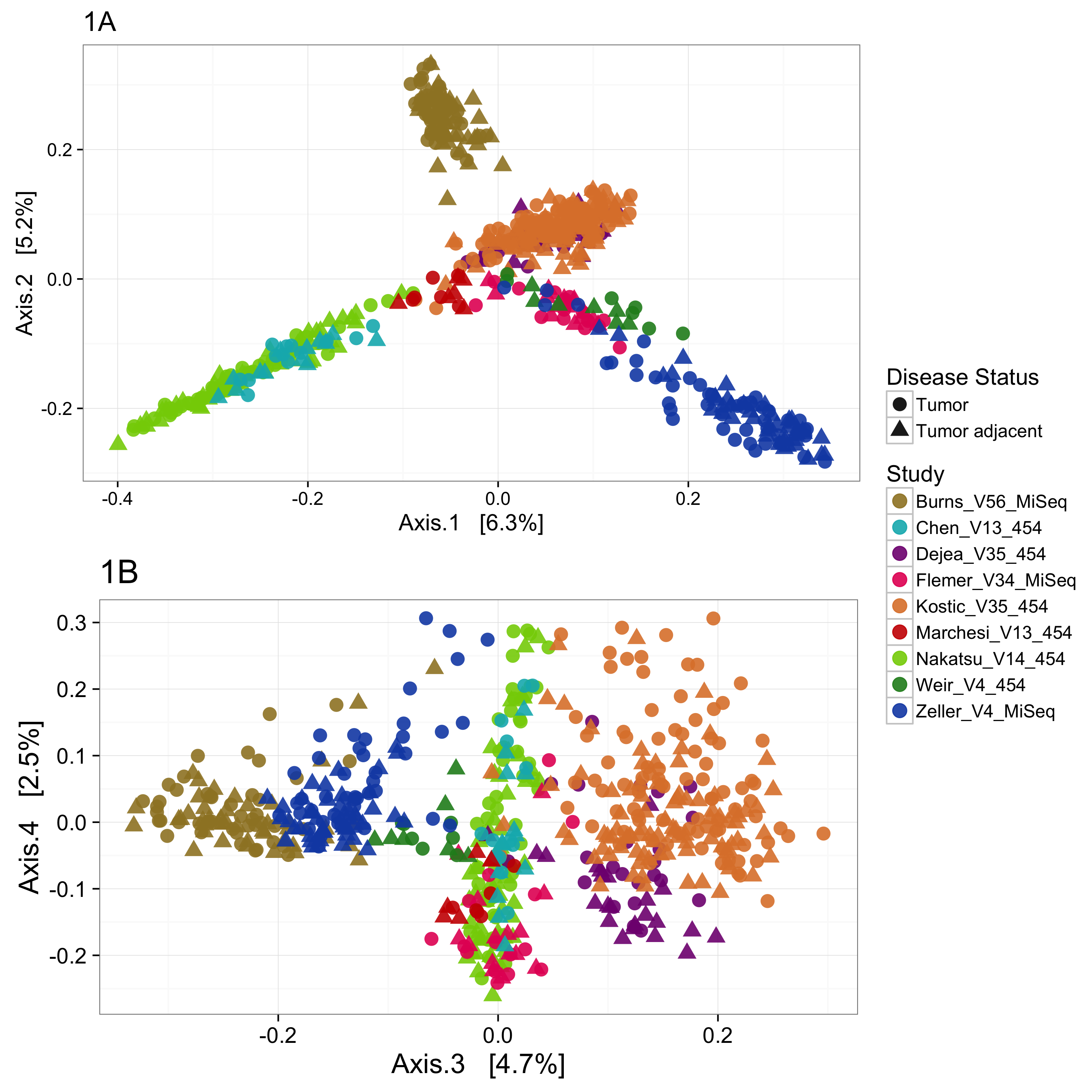

Supplement: S1 Fig — Plot points indicate individual samples, shapes indicate disease status (circle: Tumor, triangle: Tumor adjacent) and colors indicate various studies included in the meta-analysis (Target gene and sequencing platform are also incorporated in the study acronym) (A) Communities are compared in the PC1 vs PC2 axis where cohorts cluster tightly illustrating a strong study effect followed by the gene target region sequenced and (B) PC3 vs PC4 axis which resolves the study participants further. (TIFF) [file pone.0207002.s001.tiff]

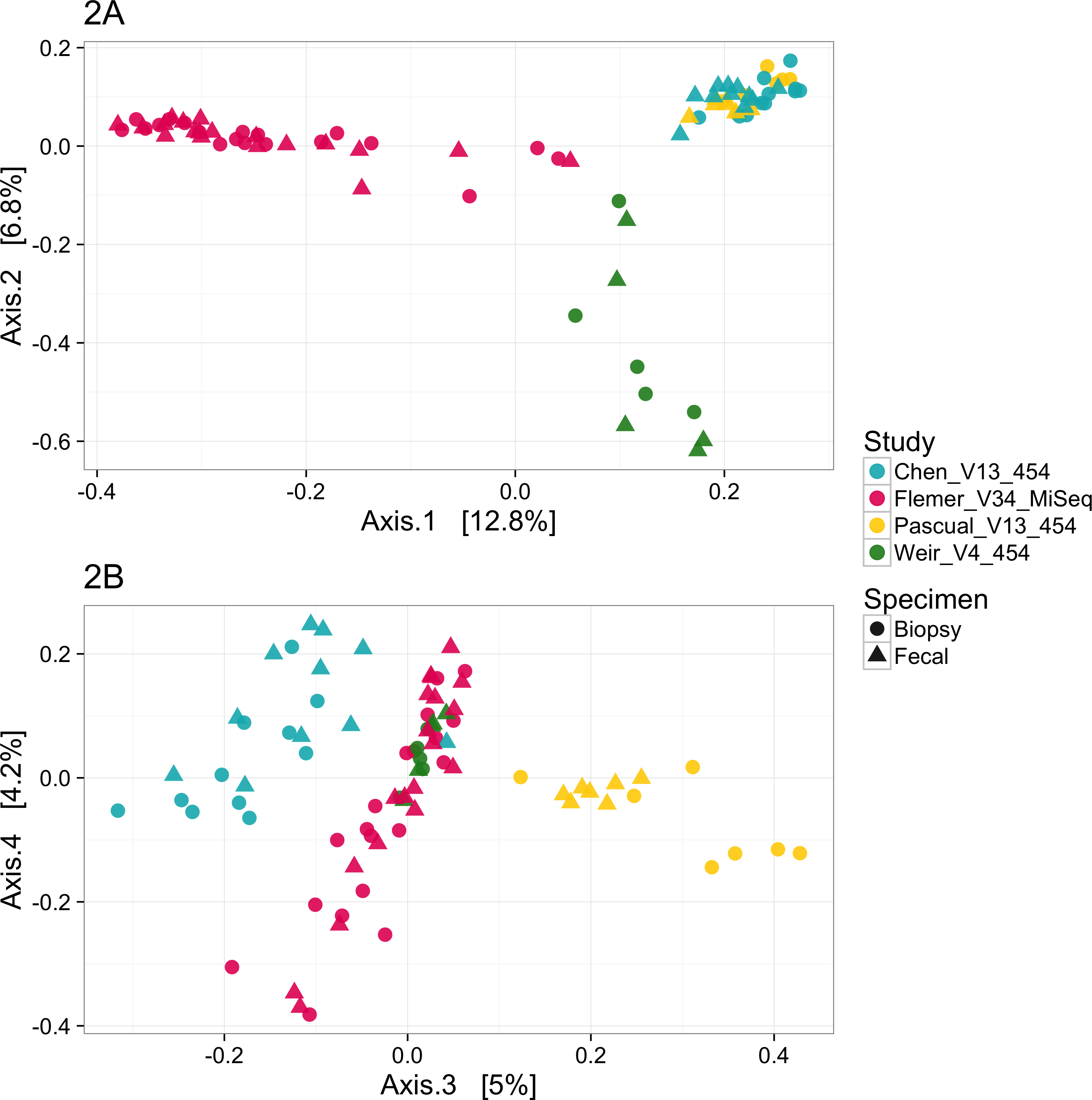

Supplement: S2 Fig — Plot points indicate individual samples, shapes indicate disease status (circle: Biopsy, CRC: Colorectal cancer) and colors indicate various studies included in the meta-analysis (Target gene and sequencing platform are also incorporated in the study acronym) (A) Communities are compared in the PC1 vs PC2 axis where cohorts cluster tightly illustrating a strong study effect followed by the gene target region sequenced and (B) PC3 vs PC4 axis which resolves the study participants further. (TIFF) [file pone.0207002.s002.tiff]
