# Supplementary material for: Re-purposing 16S rRNA gene sequence data from within case paired tumor biopsy and tumor-adjacent biopsy or fecal samples to identify microbial markers for colorectal cancer
Source: PLoS One. 2018 Nov 9;13(11):e0207002. doi: 10.1371/journal.pone.0207002 (PMC6226189; doi:10.1371/journal.pone.0207002)
Supplement: S1 Table — (DOCX) [file pone.0207002.s005.docx]

| **Study Abbreviation** | **Link to raw data** |
| --- | --- |
| Marchesi_V13_454_2011 | <http://www.cmbi.ru.nl/~dutilh/marchesi_data.zip> |
| Kostic_V35_454_2012 | <http://www.ncbi.nlm.nih.gov/Traces/study/?acc=SRP000383> |
| Chen_V13_454_2012 | <https://www.ncbi.nlm.nih.gov/sra/?term=SRP009633> |
| Geng_V12_454_2013 | <https://www.ncbi.nlm.nih.gov/Traces/study/?acc=SRP016877> |
| Weir_V4_454_2013 | <https://www.ebi.ac.uk/ena/data/view/PRJEB1415> |
| Zeller_V4_MiSeq_2014 | <https://www.ebi.ac.uk/ena/data/view/PRJEB6070> |
| Nakatsu_V14_454_2015 | <https://www.ncbi.nlm.nih.gov/sra/?term=PRJNA280026> |
| Burns_V56_MiSeq_2015 | <https://www.ncbi.nlm.nih.gov/sra/?term=PRJNA284355> |
| Pascual_V13_454_2015 | MG-RAST accession 4542409.3 through 4542468.3 facilitated by author |
| Sears_V35_454_2016 | <https://www.ncbi.nlm.nih.gov/bioproject?term=PRJNA258534> |
| Flemer_V34_MiSeq_2016 | Privately shared by author upon request by email |
