# Supplementary material for: Re-purposing 16S rRNA gene sequence data from within case paired tumor biopsy and tumor-adjacent biopsy or fecal samples to identify microbial markers for colorectal cancer
Source: PLoS One. 2018 Nov 9;13(11):e0207002. doi: 10.1371/journal.pone.0207002 (PMC6226189; doi:10.1371/journal.pone.0207002)
Supplement: S2 Table — Taxonomy follows the convention of family, genus. Abbreviations for S2 Table: LogFC: Log2Fold Change, τ2: The (total) amount of heterogeneity among the true effects, SE: Standard error, QE: Test statistic for the test of (residual) heterogeneity from the full model, QEp: p-value associated with QE, I2: For a random-effects model, I2 estimates (in percent) how much of the total variability in the effect size estimates (which is composed of heterogeneity plus sampling variability) can be attributed to heterogeneity among the true effects, H2: estimates the ratio of the total amount of variability in the effect size estimates to the amount of sampling variability, FDR: False Discovery Rate, RE:Random Effects. (DOCX) [file pone.0207002.s006.docx]

| **Taxonomy** | **Study** | **LogFC** | **CILB** | **CIUB** | **p** | **tau** | **SE_Tau2** | **QE** | **QEp** | **I2** | **H2** | **FDR** |
| --- | --- | --- | --- | --- | --- | --- | --- | --- | --- | --- | --- | --- |
| Firmicutes;unc | **RE-Model** | -0.7 | -1.1 | -0.4 | 6.4E-05 | 0 | 0.13 | 4.0 | 0.8 | 0.0 | 1.0 | 0.002 |
| Firmicutes;unc | Burns_V56_MiSeq | -1.4 | -2.6 | -0.1 |  |  |  |  |  |  |  | 0.002 |
| Firmicutes;unc | Chen_V13_454 | -0.9 | -2.1 | 0.3 |  |  |  |  |  |  |  | 0.002 |
| Firmicutes;unc | Dejea_V35_454 | -0.4 | -1.6 | 0.8 |  |  |  |  |  |  |  | 0.002 |
| Firmicutes;unc | Flemer_V34_MiSeq | -0.2 | -1.1 | 0.7 |  |  |  |  |  |  |  | 0.002 |
| Firmicutes;unc | Kostic_V35_454 | -1.0 | -1.7 | -0.2 |  |  |  |  |  |  |  | 0.002 |
| Firmicutes;unc | Marchesi_V13_454 | 0.1 | -2.0 | 2.3 |  |  |  |  |  |  |  | 0.002 |
| Firmicutes;unc | katsu_V14_454 | -0.8 | -1.9 | 0.3 |  |  |  |  |  |  |  | 0.002 |
| Firmicutes;unc | Zeller_V4_MiSeq | -0.8 | -1.5 | 0.0 |  |  |  |  |  |  |  | 0.002 |
| Firmicutes;Parvimonas | **RE-Model** | 1.5 | 0.8 | 2.3 | 8.1E-05 | 0.6485 | 0.63 | 17.3 | 0.0 | 57.2 | 2.3 | 0.002 |
| Firmicutes;Parvimonas | Burns_V56_MiSeq | 0.7 | -0.8 | 2.2 |  |  |  |  |  |  |  | 0.002 |
| Firmicutes;Parvimonas | Chen_V13_454 | 0.5 | -0.8 | 1.9 |  |  |  |  |  |  |  | 0.002 |
| Firmicutes;Parvimonas | Dejea_V35_454 | 5.1 | 2.3 | 7.9 |  |  |  |  |  |  |  | 0.002 |
| Firmicutes;Parvimonas | Flemer_V34_MiSeq | 0.5 | -0.6 | 1.7 |  |  |  |  |  |  |  | 0.002 |
| Firmicutes;Parvimonas | Kostic_V35_454 | 2.0 | 1.0 | 3.1 |  |  |  |  |  |  |  | 0.002 |
| Firmicutes;Parvimonas | Marchesi_V13_454 | 0.5 | -2.1 | 3.0 |  |  |  |  |  |  |  | 0.002 |
| Firmicutes;Parvimonas | katsu_V14_454 | 1.8 | 0.5 | 3.0 |  |  |  |  |  |  |  | 0.002 |
| Firmicutes;Parvimonas | Zeller_V4_MiSeq | 2.5 | 1.3 | 3.7 |  |  |  |  |  |  |  | 0.002 |
| Firmicutes;Faecalibacterium | **RE-Model** | -0.7 | -1.1 | -0.3 | 4.7E-04 | 2.00E-06 | 0.15 | 9.4 | 0.2 | 0.0 | 1.0 | 0.006 |
| Firmicutes;Faecalibacterium | Burns_V56_MiSeq | -0.8 | -2.2 | 0.5 |  |  |  |  |  |  |  | 0.006 |
| Firmicutes;Faecalibacterium | Chen_V13_454 | -1.5 | -2.8 | -0.2 |  |  |  |  |  |  |  | 0.006 |
| Firmicutes;Faecalibacterium | Dejea_V35_454 | -1.3 | -2.6 | 0.0 |  |  |  |  |  |  |  | 0.006 |
| Firmicutes;Faecalibacterium | Flemer_V34_MiSeq | -0.1 | -1.0 | 0.7 |  |  |  |  |  |  |  | 0.006 |
| Firmicutes;Faecalibacterium | Kostic_V35_454 | -1.0 | -1.9 | -0.1 |  |  |  |  |  |  |  | 0.006 |
| Firmicutes;Faecalibacterium | Marchesi_V13_454 | 1.8 | -0.5 | 4.1 |  |  |  |  |  |  |  | 0.006 |
| Firmicutes;Faecalibacterium | katsu_V14_454 | -0.5 | -1.8 | 0.8 |  |  |  |  |  |  |  | 0.006 |
| Firmicutes;Faecalibacterium | Zeller_V4_MiSeq | -0.7 | -1.5 | 0.1 |  |  |  |  |  |  |  | 0.006 |
| Fusobacteria;Fusobacterium | **RE-Model** | 2.6 | 1.0 | 4.3 | 2.1E-03 | 5.33507 | 3.17 | 74.4 | 0.0 | 92.0 | 12.6 | 0.022 |
| Fusobacteria;Fusobacterium | Burns_V56_MiSeq | 1.4 | 0.0 | 2.7 |  |  |  |  |  |  |  | 0.022 |
| Fusobacteria;Fusobacterium | Chen_V13_454 | 0.7 | -0.7 | 2.2 |  |  |  |  |  |  |  | 0.022 |
| Fusobacteria;Fusobacterium | Dejea_V35_454 | 7.7 | 5.8 | 9.5 |  |  |  |  |  |  |  | 0.022 |
| Fusobacteria;Fusobacterium | Flemer_V34_MiSeq | 0.2 | -0.9 | 1.2 |  |  |  |  |  |  |  | 0.022 |
| Fusobacteria;Fusobacterium | Kostic_V35_454 | 1.5 | 0.4 | 2.5 |  |  |  |  |  |  |  | 0.022 |
| Fusobacteria;Fusobacterium | Marchesi_V13_454 | 2.1 | -0.7 | 5.0 |  |  |  |  |  |  |  | 0.022 |
| Fusobacteria;Fusobacterium | katsu_V14_454 | 3.6 | 2.2 | 5.0 |  |  |  |  |  |  |  | 0.022 |
| Fusobacteria;Fusobacterium | Zeller_V4_MiSeq | 4.3 | 3.2 | 5.5 |  |  |  |  |  |  |  | 0.022 |
| Firmicutes;Peptostreptococcus | **RE-Model** | 1.0 | 0.3 | 1.8 | 7.2E-03 | 0.61718 | 0.60 | 18.9 | 0.0 | 59.0 | 2.4 | 0.059 |
| Firmicutes;Peptostreptococcus | Burns_V56_MiSeq | 0.7 | -1.0 | 2.4 |  |  |  |  |  |  |  | 0.059 |
| Firmicutes;Peptostreptococcus | Chen_V13_454 | 0.3 | -1.1 | 1.6 |  |  |  |  |  |  |  | 0.059 |
| Firmicutes;Peptostreptococcus | Dejea_V35_454 | 3.7 | 0.6 | 6.8 |  |  |  |  |  |  |  | 0.059 |
| Firmicutes;Peptostreptococcus | Flemer_V34_MiSeq | 0.1 | -0.7 | 0.8 |  |  |  |  |  |  |  | 0.059 |
| Firmicutes;Peptostreptococcus | Kostic_V35_454 | 1.2 | 0.1 | 2.3 |  |  |  |  |  |  |  | 0.059 |
| Firmicutes;Peptostreptococcus | Marchesi_V13_454 | -0.5 | -3.1 | 2.2 |  |  |  |  |  |  |  | 0.059 |
| Firmicutes;Peptostreptococcus | katsu_V14_454 | 1.4 | 0.0 | 2.8 |  |  |  |  |  |  |  | 0.059 |
| Firmicutes;Peptostreptococcus | Zeller_V4_MiSeq | 2.5 | 1.3 | 3.6 |  |  |  |  |  |  |  | 0.059 |
| Fusobacteria;Leptotrichia | **RE-Model** | 1.4 | 0.2 | 2.6 | 1.8E-02 | 0.99504 | 1.28 | 9.6 | 0.0 | 57.3 | 2.3 | 0.105 |
| Fusobacteria;Leptotrichia | Chen_V13_454 | 0.2 | -1.2 | 1.5 |  |  |  |  |  |  |  | 0.105 |
| Fusobacteria;Leptotrichia | Dejea_V35_454 | 4.1 | 1.1 | 7.2 |  |  |  |  |  |  |  | 0.105 |
| Fusobacteria;Leptotrichia | Kostic_V35_454 | 0.6 | -0.8 | 2.1 |  |  |  |  |  |  |  | 0.105 |
| Fusobacteria;Leptotrichia | katsu_V14_454 | 1.4 | -1.0 | 3.7 |  |  |  |  |  |  |  | 0.105 |
| Fusobacteria;Leptotrichia | Zeller_V4_MiSeq | 2.4 | 1.0 | 3.9 |  |  |  |  |  |  |  | 0.105 |
| Firmicutes;Streptococcus | **RE-Model** | 0.5 | 0.1 | 1.0 | 1.9E-02 | 0 | 0.22 | 5.7 | 0.6 | 0.0 | 1.0 | 0.105 |
| Firmicutes;Streptococcus | Burns_V56_MiSeq | 0.2 | -1.1 | 1.5 |  |  |  |  |  |  |  | 0.105 |
| Firmicutes;Streptococcus | Chen_V13_454 | 1.2 | -0.1 | 2.6 |  |  |  |  |  |  |  | 0.105 |
| Firmicutes;Streptococcus | Dejea_V35_454 | 0.1 | -1.7 | 1.9 |  |  |  |  |  |  |  | 0.105 |
| Firmicutes;Streptococcus | Flemer_V34_MiSeq | -0.2 | -1.3 | 1.0 |  |  |  |  |  |  |  | 0.105 |
| Firmicutes;Streptococcus | Kostic_V35_454 | 0.6 | -0.5 | 1.8 |  |  |  |  |  |  |  | 0.105 |
| Firmicutes;Streptococcus | Marchesi_V13_454 | -0.9 | -3.8 | 1.9 |  |  |  |  |  |  |  | 0.105 |
| Firmicutes;Streptococcus | katsu_V14_454 | 1.3 | 0.0 | 2.6 |  |  |  |  |  |  |  | 0.105 |
| Firmicutes;Streptococcus | Zeller_V4_MiSeq | 0.7 | -0.4 | 1.8 |  |  |  |  |  |  |  | 0.105 |
| Firmicutes;unc | **RE-Model** | 1.0 | 0.2 | 1.9 | 2.0E-02 | 0.88764 | 0.80 | 20.1 | 0.0 | 64.7 | 2.8 | 0.105 |
| Firmicutes;unc | Burns_V56_MiSeq | 0.4 | -1.2 | 2.0 |  |  |  |  |  |  |  | 0.105 |
| Firmicutes;unc | Chen_V13_454 | 0.9 | -0.7 | 2.5 |  |  |  |  |  |  |  | 0.105 |
| Firmicutes;unc | Dejea_V35_454 | 4.0 | 1.8 | 6.1 |  |  |  |  |  |  |  | 0.105 |
| Firmicutes;unc | Flemer_V34_MiSeq | 0.0 | -0.8 | 0.7 |  |  |  |  |  |  |  | 0.105 |
| Firmicutes;unc | Kostic_V35_454 | 1.1 | 0.0 | 2.2 |  |  |  |  |  |  |  | 0.105 |
| Firmicutes;unc | Marchesi_V13_454 | 0.1 | -2.6 | 2.9 |  |  |  |  |  |  |  | 0.105 |
| Firmicutes;unc | katsu_V14_454 | -0.4 | -2.7 | 2.0 |  |  |  |  |  |  |  | 0.105 |
| Firmicutes;unc | Zeller_V4_MiSeq | 2.1 | 0.9 | 3.3 |  |  |  |  |  |  |  | 0.105 |
| Firmicutes;Blautia | **RE-Model** | -0.5 | -0.9 | 0.0 | 2.8E-02 | 0 | 0.18 | 2.8 | 0.9 | 0.0 | 1.0 | 0.116 |
| Firmicutes;Blautia | Burns_V56_MiSeq | 0.0 | -1.5 | 1.5 |  |  |  |  |  |  |  | 0.116 |
| Firmicutes;Blautia | Chen_V13_454 | -0.7 | -2.1 | 0.7 |  |  |  |  |  |  |  | 0.116 |
| Firmicutes;Blautia | Dejea_V35_454 | 0.3 | -1.2 | 1.9 |  |  |  |  |  |  |  | 0.116 |
| Firmicutes;Blautia | Flemer_V34_MiSeq | -0.4 | -1.5 | 0.7 |  |  |  |  |  |  |  | 0.116 |
| Firmicutes;Blautia | Kostic_V35_454 | -0.3 | -1.3 | 0.7 |  |  |  |  |  |  |  | 0.116 |
| Firmicutes;Blautia | Marchesi_V13_454 | -0.3 | -3.0 | 2.4 |  |  |  |  |  |  |  | 0.116 |
| Firmicutes;Blautia | katsu_V14_454 | -1.0 | -2.2 | 0.2 |  |  |  |  |  |  |  | 0.116 |
| Firmicutes;Blautia | Zeller_V4_MiSeq | -0.7 | -1.5 | 0.2 |  |  |  |  |  |  |  | 0.116 |
| Firmicutes;unc | **RE-Model** | -0.4 | -0.8 | -0.1 | 2.6E-02 | 0 | 0.15 | 3.2 | 0.9 | 0.0 | 1.0 | 0.116 |
| Firmicutes;unc | Burns_V56_MiSeq | -0.2 | -1.5 | 1.1 |  |  |  |  |  |  |  | 0.116 |
| Firmicutes;unc | Chen_V13_454 | -1.1 | -2.5 | 0.3 |  |  |  |  |  |  |  | 0.116 |
| Firmicutes;unc | Dejea_V35_454 | -0.4 | -1.7 | 1.0 |  |  |  |  |  |  |  | 0.116 |
| Firmicutes;unc | Flemer_V34_MiSeq | -0.2 | -1.1 | 0.8 |  |  |  |  |  |  |  | 0.116 |
| Firmicutes;unc | Kostic_V35_454 | -0.1 | -1.0 | 0.8 |  |  |  |  |  |  |  | 0.116 |
| Firmicutes;unc | Marchesi_V13_454 | -0.3 | -2.8 | 2.2 |  |  |  |  |  |  |  | 0.116 |
| Firmicutes;unc | katsu_V14_454 | -0.3 | -1.5 | 1.0 |  |  |  |  |  |  |  | 0.116 |
| Firmicutes;unc | Zeller_V4_MiSeq | -0.8 | -1.6 | -0.1 |  |  |  |  |  |  |  | 0.116 |
| Firmicutes;Dialister | **RE-Model** | 0.5 | 0.0 | 1.0 | 4.3E-02 | 0 | 0.24 | 2.7 | 0.9 | 0.0 | 1.0 | 0.147 |
| Firmicutes;Dialister | Burns_V56_MiSeq | 0.1 | -1.4 | 1.6 |  |  |  |  |  |  |  | 0.147 |
| Firmicutes;Dialister | Chen_V13_454 | 0.3 | -1.3 | 1.9 |  |  |  |  |  |  |  | 0.147 |
| Firmicutes;Dialister | Dejea_V35_454 | 1.2 | -0.8 | 3.3 |  |  |  |  |  |  |  | 0.147 |
| Firmicutes;Dialister | Flemer_V34_MiSeq | 0.5 | -0.7 | 1.6 |  |  |  |  |  |  |  | 0.147 |
| Firmicutes;Dialister | Kostic_V35_454 | 0.8 | -0.4 | 2.0 |  |  |  |  |  |  |  | 0.147 |
| Firmicutes;Dialister | Marchesi_V13_454 | -1.0 | -3.8 | 1.8 |  |  |  |  |  |  |  | 0.147 |
| Firmicutes;Dialister | katsu_V14_454 | 0.2 | -1.1 | 1.5 |  |  |  |  |  |  |  | 0.147 |
| Firmicutes;Dialister | Zeller_V4_MiSeq | 0.8 | -0.3 | 1.8 |  |  |  |  |  |  |  | 0.147 |
| Bacteroidetes;unc | **RE-Model** | -0.4 | -0.9 | 0.0 | 4.2E-02 | 0 | 0.19 | 2.0 | 1.0 | 0.0 | 1.0 | 0.147 |
| Bacteroidetes;unc | Burns_V56_MiSeq | -0.2 | -1.7 | 1.3 |  |  |  |  |  |  |  | 0.147 |
| Bacteroidetes;unc | Chen_V13_454 | -0.6 | -2.1 | 0.9 |  |  |  |  |  |  |  | 0.147 |
| Bacteroidetes;unc | Dejea_V35_454 | -0.7 | -2.1 | 0.8 |  |  |  |  |  |  |  | 0.147 |
| Bacteroidetes;unc | Flemer_V34_MiSeq | -0.3 | -1.4 | 0.7 |  |  |  |  |  |  |  | 0.147 |
| Bacteroidetes;unc | Kostic_V35_454 | -0.1 | -1.0 | 0.9 |  |  |  |  |  |  |  | 0.147 |
| Bacteroidetes;unc | Marchesi_V13_454 | 0.1 | -2.8 | 2.9 |  |  |  |  |  |  |  | 0.147 |
| Bacteroidetes;unc | katsu_V14_454 | -0.7 | -2.4 | 1.0 |  |  |  |  |  |  |  | 0.147 |
| Bacteroidetes;unc | Zeller_V4_MiSeq | -0.8 | -1.7 | 0.1 |  |  |  |  |  |  |  | 0.147 |
| Firmicutes;Dorea | **RE-Model** | -0.4 | -0.8 | 0.0 | 4.9E-02 | 0 | 0.17 | 3.6 | 0.7 | 0.0 | 1.0 | 0.155 |
| Firmicutes;Dorea | Burns_V56_MiSeq | -1.1 | -2.6 | 0.4 |  |  |  |  |  |  |  | 0.155 |
| Firmicutes;Dorea | Chen_V13_454 | -1.1 | -2.4 | 0.3 |  |  |  |  |  |  |  | 0.155 |
| Firmicutes;Dorea | Dejea_V35_454 | 0.1 | -1.2 | 1.5 |  |  |  |  |  |  |  | 0.155 |
| Firmicutes;Dorea | Flemer_V34_MiSeq | -0.3 | -1.3 | 0.7 |  |  |  |  |  |  |  | 0.155 |
| Firmicutes;Dorea | Kostic_V35_454 | -0.4 | -1.3 | 0.5 |  |  |  |  |  |  |  | 0.155 |
| Firmicutes;Dorea | Marchesi_V13_454 | 1.0 | -1.8 | 3.8 |  |  |  |  |  |  |  | 0.155 |
| Firmicutes;Dorea | Zeller_V4_MiSeq | -0.4 | -1.2 | 0.4 |  |  |  |  |  |  |  | 0.155 |
| Bacteroidetes;Parabacteroides | **RE-Model** | -0.4 | -0.8 | 0.0 | 6.6E-02 | 2.55E-06 | 0.15 | 12.3 | 0.1 | 0.0 | 1.0 | 0.193 |
| Bacteroidetes;Parabacteroides | Burns_V56_MiSeq | -1.0 | -2.4 | 0.4 |  |  |  |  |  |  |  | 0.193 |
| Bacteroidetes;Parabacteroides | Chen_V13_454 | -0.4 | -1.9 | 1.1 |  |  |  |  |  |  |  | 0.193 |
| Bacteroidetes;Parabacteroides | Dejea_V35_454 | -0.6 | -2.0 | 0.8 |  |  |  |  |  |  |  | 0.193 |
| Bacteroidetes;Parabacteroides | Flemer_V34_MiSeq | -0.2 | -1.1 | 0.6 |  |  |  |  |  |  |  | 0.193 |
| Bacteroidetes;Parabacteroides | Kostic_V35_454 | -0.3 | -1.2 | 0.5 |  |  |  |  |  |  |  | 0.193 |
| Bacteroidetes;Parabacteroides | Marchesi_V13_454 | 3.7 | 0.9 | 6.4 |  |  |  |  |  |  |  | 0.193 |
| Bacteroidetes;Parabacteroides | katsu_V14_454 | 0.4 | -0.9 | 1.7 |  |  |  |  |  |  |  | 0.193 |
| Bacteroidetes;Parabacteroides | Zeller_V4_MiSeq | -0.8 | -1.6 | 0.0 |  |  |  |  |  |  |  | 0.193 |
| Firmicutes;Phascolarctobacterium | **RE-Model** | -0.4 | -0.9 | 0.1 | 1.1E-01 | 0 | 0.23 | 2.6 | 0.8 | 0.0 | 1.0 | 0.307 |
| Firmicutes;Phascolarctobacterium | Burns_V56_MiSeq | 0.0 | -1.4 | 1.5 |  |  |  |  |  |  |  | 0.307 |
| Firmicutes;Phascolarctobacterium | Chen_V13_454 | -0.3 | -1.9 | 1.2 |  |  |  |  |  |  |  | 0.307 |
| Firmicutes;Phascolarctobacterium | Dejea_V35_454 | -0.1 | -1.5 | 1.4 |  |  |  |  |  |  |  | 0.307 |
| Firmicutes;Phascolarctobacterium | Flemer_V34_MiSeq | -0.1 | -1.2 | 1.1 |  |  |  |  |  |  |  | 0.307 |
| Firmicutes;Phascolarctobacterium | Kostic_V35_454 | -0.3 | -1.5 | 0.8 |  |  |  |  |  |  |  | 0.307 |
| Firmicutes;Phascolarctobacterium | Zeller_V4_MiSeq | -1.0 | -2.0 | -0.1 |  |  |  |  |  |  |  | 0.307 |
| Proteobacteria;Campylobacter | **RE-Model** | 0.6 | -0.2 | 1.5 | 1.3E-01 | 0.27958 | 0.61 | 5.6 | 0.2 | 32.4 | 1.5 | 0.307 |
| Proteobacteria;Campylobacter | Chen_V13_454 | 0.4 | -1.0 | 1.7 |  |  |  |  |  |  |  | 0.307 |
| Proteobacteria;Campylobacter | Flemer_V34_MiSeq | 0.0 | -0.7 | 0.7 |  |  |  |  |  |  |  | 0.307 |
| Proteobacteria;Campylobacter | Marchesi_V13_454 | 2.1 | -0.7 | 4.9 |  |  |  |  |  |  |  | 0.307 |
| Proteobacteria;Campylobacter | katsu_V14_454 | 2.2 | -0.1 | 4.6 |  |  |  |  |  |  |  | 0.307 |
| Proteobacteria;Campylobacter | Zeller_V4_MiSeq | 1.0 | -0.9 | 2.9 |  |  |  |  |  |  |  | 0.307 |
| Firmicutes;Bulleidia | **RE-Model** | 0.6 | -0.2 | 1.4 | 1.3E-01 | 0.38615 | 0.58 | 8.1 | 0.1 | 43.8 | 1.8 | 0.307 |
| Firmicutes;Bulleidia | Burns_V56_MiSeq | 0.0 | -1.5 | 1.5 |  |  |  |  |  |  |  | 0.307 |
| Firmicutes;Bulleidia | Chen_V13_454 | 0.1 | -1.3 | 1.6 |  |  |  |  |  |  |  | 0.307 |
| Firmicutes;Bulleidia | Flemer_V34_MiSeq | 0.1 | -0.6 | 0.8 |  |  |  |  |  |  |  | 0.307 |
| Firmicutes;Bulleidia | Marchesi_V13_454 | 1.0 | -1.8 | 3.7 |  |  |  |  |  |  |  | 0.307 |
| Firmicutes;Bulleidia | katsu_V14_454 | 1.3 | -1.0 | 3.6 |  |  |  |  |  |  |  | 0.307 |
| Firmicutes;Bulleidia | Zeller_V4_MiSeq | 2.1 | 0.7 | 3.5 |  |  |  |  |  |  |  | 0.307 |
| Firmicutes;unc | **RE-Model** | -0.3 | -0.8 | 0.1 | 1.5E-01 | 0 | 0.21 | 4.8 | 0.6 | 0.0 | 1.0 | 0.307 |
| Firmicutes;unc | Burns_V56_MiSeq | -0.8 | -2.4 | 0.7 |  |  |  |  |  |  |  | 0.307 |
| Firmicutes;unc | Chen_V13_454 | -0.1 | -1.7 | 1.5 |  |  |  |  |  |  |  | 0.307 |
| Firmicutes;unc | Dejea_V35_454 | 0.6 | -1.2 | 2.3 |  |  |  |  |  |  |  | 0.307 |
| Firmicutes;unc | Flemer_V34_MiSeq | 0.0 | -1.0 | 1.0 |  |  |  |  |  |  |  | 0.307 |
| Firmicutes;unc | Kostic_V35_454 | -1.0 | -2.0 | 0.0 |  |  |  |  |  |  |  | 0.307 |
| Firmicutes;unc | Marchesi_V13_454 | 0.9 | -1.8 | 3.7 |  |  |  |  |  |  |  | 0.307 |
| Firmicutes;unc | Zeller_V4_MiSeq | -0.3 | -1.2 | 0.6 |  |  |  |  |  |  |  | 0.307 |
| Firmicutes;[Ruminococcus] | **RE-Model** | -0.3 | -0.8 | 0.1 | 1.5E-01 | 0 | 0.19 | 1.3 | 1.0 | 0.0 | 1.0 | 0.307 |
| Firmicutes;[Ruminococcus] | Burns_V56_MiSeq | -1.0 | -2.5 | 0.6 |  |  |  |  |  |  |  | 0.307 |
| Firmicutes;[Ruminococcus] | Chen_V13_454 | -0.2 | -1.8 | 1.4 |  |  |  |  |  |  |  | 0.307 |
| Firmicutes;[Ruminococcus] | Dejea_V35_454 | 0.0 | -1.2 | 1.1 |  |  |  |  |  |  |  | 0.307 |
| Firmicutes;[Ruminococcus] | Flemer_V34_MiSeq | -0.1 | -1.2 | 0.9 |  |  |  |  |  |  |  | 0.307 |
| Firmicutes;[Ruminococcus] | Kostic_V35_454 | -0.3 | -1.3 | 0.6 |  |  |  |  |  |  |  | 0.307 |
| Firmicutes;[Ruminococcus] | Marchesi_V13_454 | -0.1 | -2.8 | 2.5 |  |  |  |  |  |  |  | 0.307 |
| Firmicutes;[Ruminococcus] | Zeller_V4_MiSeq | -0.5 | -1.4 | 0.4 |  |  |  |  |  |  |  | 0.307 |
| Proteobacteria;Sutterella | **RE-Model** | -0.4 | -0.9 | 0.1 | 1.4E-01 | 0.0946 | 0.27 | 6.6 | 0.5 | 17.5 | 1.2 | 0.307 |
| Proteobacteria;Sutterella | Burns_V56_MiSeq | 0.5 | -1.1 | 2.1 |  |  |  |  |  |  |  | 0.307 |
| Proteobacteria;Sutterella | Chen_V13_454 | -0.2 | -1.8 | 1.4 |  |  |  |  |  |  |  | 0.307 |
| Proteobacteria;Sutterella | Dejea_V35_454 | -0.1 | -1.6 | 1.3 |  |  |  |  |  |  |  | 0.307 |
| Proteobacteria;Sutterella | Flemer_V34_MiSeq | 0.1 | -0.9 | 1.2 |  |  |  |  |  |  |  | 0.307 |
| Proteobacteria;Sutterella | Kostic_V35_454 | -1.4 | -2.4 | -0.4 |  |  |  |  |  |  |  | 0.307 |
| Proteobacteria;Sutterella | Marchesi_V13_454 | -0.2 | -2.8 | 2.4 |  |  |  |  |  |  |  | 0.307 |
| Proteobacteria;Sutterella | katsu_V14_454 | -0.1 | -2.4 | 2.3 |  |  |  |  |  |  |  | 0.307 |
| Proteobacteria;Sutterella | Zeller_V4_MiSeq | -0.6 | -1.5 | 0.3 |  |  |  |  |  |  |  | 0.307 |
| Firmicutes;Veillonella | **RE-Model** | 0.4 | -0.2 | 1.1 | 1.9E-01 | 5.84E-07 | 0.41 | 6.4 | 0.3 | 0.0 | 1.0 | 0.364 |
| Firmicutes;Veillonella | Burns_V56_MiSeq | 0.3 | -1.4 | 1.9 |  |  |  |  |  |  |  | 0.364 |
| Firmicutes;Veillonella | Chen_V13_454 | 0.0 | -1.6 | 1.6 |  |  |  |  |  |  |  | 0.364 |
| Firmicutes;Veillonella | Dejea_V35_454 | 3.3 | 0.2 | 6.4 |  |  |  |  |  |  |  | 0.364 |
| Firmicutes;Veillonella | Kostic_V35_454 | -0.3 | -1.8 | 1.1 |  |  |  |  |  |  |  | 0.364 |
| Firmicutes;Veillonella | Marchesi_V13_454 | 2.1 | -0.7 | 5.0 |  |  |  |  |  |  |  | 0.364 |
| Firmicutes;Veillonella | Zeller_V4_MiSeq | 0.6 | -0.6 | 1.8 |  |  |  |  |  |  |  | 0.364 |
| Firmicutes;Ruminococcus | **RE-Model** | -0.5 | -1.3 | 0.3 | 2.0E-01 | 0.42458 | 0.56 | 9.4 | 0.1 | 49.6 | 2.0 | 0.371 |
| Firmicutes;Ruminococcus | Burns_V56_MiSeq | 0.1 | -1.4 | 1.5 |  |  |  |  |  |  |  | 0.371 |
| Firmicutes;Ruminococcus | Chen_V13_454 | 0.1 | -1.5 | 1.7 |  |  |  |  |  |  |  | 0.371 |
| Firmicutes;Ruminococcus | Dejea_V35_454 | 0.2 | -2.8 | 3.3 |  |  |  |  |  |  |  | 0.371 |
| Firmicutes;Ruminococcus | Flemer_V34_MiSeq | -0.1 | -1.3 | 1.0 |  |  |  |  |  |  |  | 0.371 |
| Firmicutes;Ruminococcus | Kostic_V35_454 | -2.0 | -3.1 | -0.9 |  |  |  |  |  |  |  | 0.371 |
| Firmicutes;Ruminococcus | Zeller_V4_MiSeq | -0.4 | -1.3 | 0.5 |  |  |  |  |  |  |  | 0.371 |
| Bacteroidetes;Porphyromos | **RE-Model** | 0.4 | -0.3 | 1.0 | 2.8E-01 | 0.21496 | 0.41 | 12.3 | 0.1 | 30.2 | 1.4 | 0.492 |
| Bacteroidetes;Porphyromos | Burns_V56_MiSeq | -0.4 | -1.9 | 1.2 |  |  |  |  |  |  |  | 0.492 |
| Bacteroidetes;Porphyromos | Chen_V13_454 | 0.5 | -1.1 | 2.0 |  |  |  |  |  |  |  | 0.492 |
| Bacteroidetes;Porphyromos | Dejea_V35_454 | 4.1 | 1.1 | 7.2 |  |  |  |  |  |  |  | 0.492 |
| Bacteroidetes;Porphyromos | Flemer_V34_MiSeq | 0.0 | -0.7 | 0.7 |  |  |  |  |  |  |  | 0.492 |
| Bacteroidetes;Porphyromos | Kostic_V35_454 | 0.1 | -1.2 | 1.3 |  |  |  |  |  |  |  | 0.492 |
| Bacteroidetes;Porphyromos | Marchesi_V13_454 | -1.4 | -4.1 | 1.3 |  |  |  |  |  |  |  | 0.492 |
| Bacteroidetes;Porphyromos | Zeller_V4_MiSeq | 1.5 | 0.1 | 2.8 |  |  |  |  |  |  |  | 0.492 |
| Bacteroidetes;unc | **RE-Model** | -0.2 | -0.7 | 0.2 | 3.2E-01 | 0 | 0.22 | 3.1 | 0.9 | 0.0 | 1.0 | 0.534 |
| Bacteroidetes;unc | Burns_V56_MiSeq | -0.9 | -2.3 | 0.5 |  |  |  |  |  |  |  | 0.534 |
| Bacteroidetes;unc | Chen_V13_454 | 0.0 | -1.5 | 1.4 |  |  |  |  |  |  |  | 0.534 |
| Bacteroidetes;unc | Dejea_V35_454 | -1.0 | -3.1 | 1.2 |  |  |  |  |  |  |  | 0.534 |
| Bacteroidetes;unc | Flemer_V34_MiSeq | 0.0 | -1.1 | 1.0 |  |  |  |  |  |  |  | 0.534 |
| Bacteroidetes;unc | Kostic_V35_454 | 0.2 | -0.9 | 1.4 |  |  |  |  |  |  |  | 0.534 |
| Bacteroidetes;unc | Marchesi_V13_454 | -1.5 | -4.3 | 1.3 |  |  |  |  |  |  |  | 0.534 |
| Bacteroidetes;unc | katsu_V14_454 | 0.1 | -2.3 | 2.4 |  |  |  |  |  |  |  | 0.534 |
| Bacteroidetes;unc | Zeller_V4_MiSeq | -0.3 | -1.2 | 0.6 |  |  |  |  |  |  |  | 0.534 |
| Bacteroidetes;Prevotella | **RE-Model** | 0.2 | -0.2 | 0.7 | 3.3E-01 | 0 | 0.23 | 4.4 | 0.7 | 0.0 | 1.0 | 0.534 |
| Bacteroidetes;Prevotella | Burns_V56_MiSeq | 0.7 | -0.8 | 2.3 |  |  |  |  |  |  |  | 0.534 |
| Bacteroidetes;Prevotella | Chen_V13_454 | -0.8 | -2.2 | 0.6 |  |  |  |  |  |  |  | 0.534 |
| Bacteroidetes;Prevotella | Dejea_V35_454 | 0.4 | -1.3 | 2.1 |  |  |  |  |  |  |  | 0.534 |
| Bacteroidetes;Prevotella | Flemer_V34_MiSeq | 0.0 | -1.1 | 1.2 |  |  |  |  |  |  |  | 0.534 |
| Bacteroidetes;Prevotella | Kostic_V35_454 | 0.7 | -0.4 | 1.7 |  |  |  |  |  |  |  | 0.534 |
| Bacteroidetes;Prevotella | Marchesi_V13_454 | 1.3 | -1.3 | 3.9 |  |  |  |  |  |  |  | 0.534 |
| Bacteroidetes;Prevotella | katsu_V14_454 | -0.2 | -2.5 | 2.1 |  |  |  |  |  |  |  | 0.534 |
| Bacteroidetes;Prevotella | Zeller_V4_MiSeq | 0.2 | -0.8 | 1.3 |  |  |  |  |  |  |  | 0.534 |
| Firmicutes;Roseburia | **RE-Model** | -0.2 | -0.6 | 0.2 | 3.8E-01 | 0 | 0.17 | 2.9 | 0.9 | 0.0 | 1.0 | 0.594 |
| Firmicutes;Roseburia | Burns_V56_MiSeq | 0.3 | -1.2 | 1.8 |  |  |  |  |  |  |  | 0.594 |
| Firmicutes;Roseburia | Chen_V13_454 | -0.9 | -2.1 | 0.4 |  |  |  |  |  |  |  | 0.594 |
| Firmicutes;Roseburia | Dejea_V35_454 | 0.2 | -1.3 | 1.7 |  |  |  |  |  |  |  | 0.594 |
| Firmicutes;Roseburia | Flemer_V34_MiSeq | 0.0 | -0.7 | 0.7 |  |  |  |  |  |  |  | 0.594 |
| Firmicutes;Roseburia | Kostic_V35_454 | -0.5 | -1.5 | 0.5 |  |  |  |  |  |  |  | 0.594 |
| Firmicutes;Roseburia | Marchesi_V13_454 | -0.5 | -3.3 | 2.4 |  |  |  |  |  |  |  | 0.594 |
| Firmicutes;Roseburia | katsu_V14_454 | 0.5 | -1.9 | 2.8 |  |  |  |  |  |  |  | 0.594 |
| Firmicutes;Roseburia | Zeller_V4_MiSeq | -0.3 | -1.3 | 0.7 |  |  |  |  |  |  |  | 0.594 |
| Proteobacteria;unc | **RE-Model** | -0.2 | -0.6 | 0.3 | 5.3E-01 | 0 | 0.21 | 1.4 | 0.9 | 0.0 | 1.0 | 0.705 |
| Proteobacteria;unc | Burns_V56_MiSeq | -0.1 | -1.6 | 1.3 |  |  |  |  |  |  |  | 0.705 |
| Proteobacteria;unc | Chen_V13_454 | 0.3 | -1.3 | 1.8 |  |  |  |  |  |  |  | 0.705 |
| Proteobacteria;unc | Dejea_V35_454 | -0.7 | -3.7 | 2.4 |  |  |  |  |  |  |  | 0.705 |
| Proteobacteria;unc | Flemer_V34_MiSeq | 0.0 | -0.7 | 0.8 |  |  |  |  |  |  |  | 0.705 |
| Proteobacteria;unc | Kostic_V35_454 | -0.5 | -1.8 | 0.8 |  |  |  |  |  |  |  | 0.705 |
| Proteobacteria;unc | Zeller_V4_MiSeq | -0.5 | -1.5 | 0.6 |  |  |  |  |  |  |  | 0.705 |
| Firmicutes;unc | **RE-Model** | 0.2 | -0.4 | 0.9 | 5.3E-01 | 0.38451 | 0.45 | 12.8 | 0.1 | 46.9 | 1.9 | 0.705 |
| Firmicutes;unc | Burns_V56_MiSeq | -0.1 | -1.6 | 1.4 |  |  |  |  |  |  |  | 0.705 |
| Firmicutes;unc | Chen_V13_454 | 0.3 | -1.1 | 1.7 |  |  |  |  |  |  |  | 0.705 |
| Firmicutes;unc | Dejea_V35_454 | 1.3 | -1.8 | 4.3 |  |  |  |  |  |  |  | 0.705 |
| Firmicutes;unc | Flemer_V34_MiSeq | -0.5 | -1.6 | 0.6 |  |  |  |  |  |  |  | 0.705 |
| Firmicutes;unc | Kostic_V35_454 | -0.2 | -1.3 | 0.9 |  |  |  |  |  |  |  | 0.705 |
| Firmicutes;unc | Marchesi_V13_454 | 1.7 | -0.8 | 4.3 |  |  |  |  |  |  |  | 0.705 |
| Firmicutes;unc | katsu_V14_454 | 1.6 | 0.5 | 2.8 |  |  |  |  |  |  |  | 0.705 |
| Firmicutes;unc | Zeller_V4_MiSeq | -0.5 | -1.4 | 0.4 |  |  |  |  |  |  |  | 0.705 |
| Bacteroidetes;Bacteroides | **RE-Model** | 0.2 | -0.3 | 0.6 | 4.9E-01 | 0.15922 | 0.23 | 11.0 | 0.1 | 37.4 | 1.6 | 0.705 |
| Bacteroidetes;Bacteroides | Burns_V56_MiSeq | -0.1 | -1.2 | 1.1 |  |  |  |  |  |  |  | 0.705 |
| Bacteroidetes;Bacteroides | Chen_V13_454 | -0.4 | -1.5 | 0.7 |  |  |  |  |  |  |  | 0.705 |
| Bacteroidetes;Bacteroides | Dejea_V35_454 | -0.6 | -1.8 | 0.5 |  |  |  |  |  |  |  | 0.705 |
| Bacteroidetes;Bacteroides | Flemer_V34_MiSeq | 0.0 | -0.9 | 0.9 |  |  |  |  |  |  |  | 0.705 |
| Bacteroidetes;Bacteroides | Kostic_V35_454 | 1.1 | 0.3 | 1.8 |  |  |  |  |  |  |  | 0.705 |
| Bacteroidetes;Bacteroides | Marchesi_V13_454 | 1.3 | -1.0 | 3.7 |  |  |  |  |  |  |  | 0.705 |
| Bacteroidetes;Bacteroides | katsu_V14_454 | 0.7 | -0.4 | 1.8 |  |  |  |  |  |  |  | 0.705 |
| Bacteroidetes;Bacteroides | Zeller_V4_MiSeq | -0.2 | -0.9 | 0.6 |  |  |  |  |  |  |  | 0.705 |
| Firmicutes;Oscillospira | **RE-Model** | -0.1 | -0.6 | 0.3 | 5.3E-01 | 0 | 0.18 | 1.3 | 1.0 | 0.0 | 1.0 | 0.705 |
| Firmicutes;Oscillospira | Burns_V56_MiSeq | -0.1 | -1.6 | 1.4 |  |  |  |  |  |  |  | 0.705 |
| Firmicutes;Oscillospira | Chen_V13_454 | -0.3 | -1.9 | 1.2 |  |  |  |  |  |  |  | 0.705 |
| Firmicutes;Oscillospira | Dejea_V35_454 | 0.2 | -1.1 | 1.5 |  |  |  |  |  |  |  | 0.705 |
| Firmicutes;Oscillospira | Flemer_V34_MiSeq | -0.3 | -1.3 | 0.6 |  |  |  |  |  |  |  | 0.705 |
| Firmicutes;Oscillospira | Kostic_V35_454 | -0.1 | -1.0 | 0.8 |  |  |  |  |  |  |  | 0.705 |
| Firmicutes;Oscillospira | Marchesi_V13_454 | -1.1 | -3.8 | 1.6 |  |  |  |  |  |  |  | 0.705 |
| Firmicutes;Oscillospira | katsu_V14_454 | 0.3 | -1.2 | 1.8 |  |  |  |  |  |  |  | 0.705 |
| Firmicutes;Oscillospira | Zeller_V4_MiSeq | -0.2 | -1.1 | 0.8 |  |  |  |  |  |  |  | 0.705 |
| Firmicutes;Lachnobacterium | **RE-Model** | -0.2 | -0.8 | 0.4 | 5.3E-01 | 0 | 0.33 | 3.0 | 0.7 | 0.0 | 1.0 | 0.705 |
| Firmicutes;Lachnobacterium | Burns_V56_MiSeq | -0.3 | -1.7 | 1.2 |  |  |  |  |  |  |  | 0.705 |
| Firmicutes;Lachnobacterium | Chen_V13_454 | 0.6 | -1.0 | 2.2 |  |  |  |  |  |  |  | 0.705 |
| Firmicutes;Lachnobacterium | Dejea_V35_454 | -1.5 | -4.5 | 1.6 |  |  |  |  |  |  |  | 0.705 |
| Firmicutes;Lachnobacterium | Flemer_V34_MiSeq | 0.2 | -1.0 | 1.3 |  |  |  |  |  |  |  | 0.705 |
| Firmicutes;Lachnobacterium | Marchesi_V13_454 | -0.2 | -3.0 | 2.6 |  |  |  |  |  |  |  | 0.705 |
| Firmicutes;Lachnobacterium | Zeller_V4_MiSeq | -0.7 | -1.8 | 0.4 |  |  |  |  |  |  |  | 0.705 |
| Bacteroidetes;[Prevotella] | **RE-Model** | 0.2 | -0.4 | 0.7 | 5.6E-01 | 0 | 0.28 | 1.1 | 0.9 | 0.0 | 1.0 | 0.713 |
| Bacteroidetes;[Prevotella] | Burns_V56_MiSeq | 0.2 | -1.3 | 1.6 |  |  |  |  |  |  |  | 0.713 |
| Bacteroidetes;[Prevotella] | Dejea_V35_454 | 1.1 | -1.9 | 4.1 |  |  |  |  |  |  |  | 0.713 |
| Bacteroidetes;[Prevotella] | Flemer_V34_MiSeq | 0.0 | -0.7 | 0.7 |  |  |  |  |  |  |  | 0.713 |
| Bacteroidetes;[Prevotella] | Kostic_V35_454 | 0.5 | -0.8 | 1.8 |  |  |  |  |  |  |  | 0.713 |
| Bacteroidetes;[Prevotella] | Zeller_V4_MiSeq | 0.5 | -1.4 | 2.4 |  |  |  |  |  |  |  | 0.713 |
| Proteobacteria;Escherichia | **RE-Model** | 0.2 | -0.7 | 1.0 | 7.4E-01 | 0 | 0.71 | 2.2 | 0.7 | 0.0 | 1.0 | 0.798 |
| Proteobacteria;Escherichia | Chen_V13_454 | 0.8 | -0.8 | 2.4 |  |  |  |  |  |  |  | 0.798 |
| Proteobacteria;Escherichia | Dejea_V35_454 | 0.7 | -1.7 | 3.1 |  |  |  |  |  |  |  | 0.798 |
| Proteobacteria;Escherichia | Marchesi_V13_454 | -0.5 | -3.0 | 2.0 |  |  |  |  |  |  |  | 0.798 |
| Proteobacteria;Escherichia | katsu_V14_454 | -0.9 | -2.8 | 1.1 |  |  |  |  |  |  |  | 0.798 |
| Proteobacteria;Escherichia | Zeller_V4_MiSeq | 0.3 | -1.7 | 2.2 |  |  |  |  |  |  |  | 0.798 |
| Firmicutes;unc | **RE-Model** | 0.1 | -0.3 | 0.5 | 7.5E-01 | 0.03087 | 0.17 | 6.2 | 0.5 | 8.8 | 1.1 | 0.798 |
| Firmicutes;unc | Burns_V56_MiSeq | -0.6 | -1.9 | 0.7 |  |  |  |  |  |  |  | 0.798 |
| Firmicutes;unc | Chen_V13_454 | -0.4 | -2.0 | 1.2 |  |  |  |  |  |  |  | 0.798 |
| Firmicutes;unc | Dejea_V35_454 | 0.5 | -0.6 | 1.6 |  |  |  |  |  |  |  | 0.798 |
| Firmicutes;unc | Flemer_V34_MiSeq | -0.2 | -1.2 | 0.8 |  |  |  |  |  |  |  | 0.798 |
| Firmicutes;unc | Kostic_V35_454 | 0.6 | -0.2 | 1.4 |  |  |  |  |  |  |  | 0.798 |
| Firmicutes;unc | Marchesi_V13_454 | 1.3 | -1.4 | 4.0 |  |  |  |  |  |  |  | 0.798 |
| Firmicutes;unc | katsu_V14_454 | 0.3 | -1.3 | 1.8 |  |  |  |  |  |  |  | 0.798 |
| Firmicutes;unc | Zeller_V4_MiSeq | -0.3 | -1.1 | 0.5 |  |  |  |  |  |  |  | 0.798 |
| Verrucomicrobia;Akkermansia | **RE-Model** | -0.1 | -0.6 | 0.4 | 7.1E-01 | 0 | 0.23 | 0.9 | 0.9 | 0.0 | 1.0 | 0.798 |
| Verrucomicrobia;Akkermansia | Burns_V56_MiSeq | 0.3 | -1.2 | 1.8 |  |  |  |  |  |  |  | 0.798 |
| Verrucomicrobia;Akkermansia | Chen_V13_454 | -0.1 | -1.7 | 1.5 |  |  |  |  |  |  |  | 0.798 |
| Verrucomicrobia;Akkermansia | Flemer_V34_MiSeq | 0.0 | -0.7 | 0.8 |  |  |  |  |  |  |  | 0.798 |
| Verrucomicrobia;Akkermansia | Kostic_V35_454 | -0.1 | -1.5 | 1.4 |  |  |  |  |  |  |  | 0.798 |
| Verrucomicrobia;Akkermansia | Zeller_V4_MiSeq | -0.5 | -1.6 | 0.6 |  |  |  |  |  |  |  | 0.798 |
| Firmicutes;Clostridium | **RE-Model** | -0.1 | -0.6 | 0.4 | 6.5E-01 | 3.93E-06 | 0.29 | 5.2 | 0.6 | 0.0 | 1.0 | 0.798 |
| Firmicutes;Clostridium | Burns_V56_MiSeq | -0.1 | -1.6 | 1.3 |  |  |  |  |  |  |  | 0.798 |
| Firmicutes;Clostridium | Chen_V13_454 | 0.0 | -1.6 | 1.5 |  |  |  |  |  |  |  | 0.798 |
| Firmicutes;Clostridium | Dejea_V35_454 | -2.9 | -6.0 | 0.2 |  |  |  |  |  |  |  | 0.798 |
| Firmicutes;Clostridium | Flemer_V34_MiSeq | -0.3 | -1.5 | 0.8 |  |  |  |  |  |  |  | 0.798 |
| Firmicutes;Clostridium | Kostic_V35_454 | -0.1 | -1.5 | 1.4 |  |  |  |  |  |  |  | 0.798 |
| Firmicutes;Clostridium | Marchesi_V13_454 | -1.2 | -4.0 | 1.5 |  |  |  |  |  |  |  | 0.798 |
| Firmicutes;Clostridium | katsu_V14_454 | 0.3 | -1.1 | 1.8 |  |  |  |  |  |  |  | 0.798 |
| Firmicutes;Clostridium | Zeller_V4_MiSeq | 0.4 | -0.9 | 1.7 |  |  |  |  |  |  |  | 0.798 |
| Proteobacteria;Desulfovibrio | **RE-Model** | -0.1 | -0.6 | 0.4 | 6.8E-01 | 0 | 0.28 | 1.8 | 0.9 | 0.0 | 1.0 | 0.798 |
| Proteobacteria;Desulfovibrio | Burns_V56_MiSeq | 0.0 | -1.5 | 1.4 |  |  |  |  |  |  |  | 0.798 |
| Proteobacteria;Desulfovibrio | Chen_V13_454 | -0.1 | -1.5 | 1.4 |  |  |  |  |  |  |  | 0.798 |
| Proteobacteria;Desulfovibrio | Flemer_V34_MiSeq | 0.0 | -0.7 | 0.7 |  |  |  |  |  |  |  | 0.798 |
| Proteobacteria;Desulfovibrio | Marchesi_V13_454 | -1.9 | -4.8 | 0.9 |  |  |  |  |  |  |  | 0.798 |
| Proteobacteria;Desulfovibrio | katsu_V14_454 | 0.0 | -2.3 | 2.4 |  |  |  |  |  |  |  | 0.798 |
| Proteobacteria;Desulfovibrio | Zeller_V4_MiSeq | -0.3 | -1.9 | 1.3 |  |  |  |  |  |  |  | 0.798 |
| Proteobacteria;Haemophilus | **RE-Model** | -0.1 | -0.6 | 0.4 | 7.6E-01 | 0 | 0.27 | 2.7 | 0.9 | 0.0 | 1.0 | 0.798 |
| Proteobacteria;Haemophilus | Burns_V56_MiSeq | 0.0 | -1.4 | 1.5 |  |  |  |  |  |  |  | 0.798 |
| Proteobacteria;Haemophilus | Chen_V13_454 | -0.2 | -1.8 | 1.4 |  |  |  |  |  |  |  | 0.798 |
| Proteobacteria;Haemophilus | Dejea_V35_454 | -0.1 | -3.2 | 3.0 |  |  |  |  |  |  |  | 0.798 |
| Proteobacteria;Haemophilus | Flemer_V34_MiSeq | -0.3 | -1.4 | 0.8 |  |  |  |  |  |  |  | 0.798 |
| Proteobacteria;Haemophilus | Kostic_V35_454 | -0.3 | -1.8 | 1.1 |  |  |  |  |  |  |  | 0.798 |
| Proteobacteria;Haemophilus | Marchesi_V13_454 | 1.3 | -1.2 | 3.8 |  |  |  |  |  |  |  | 0.798 |
| Proteobacteria;Haemophilus | katsu_V14_454 | 0.6 | -0.9 | 2.0 |  |  |  |  |  |  |  | 0.798 |
| Proteobacteria;Haemophilus | Zeller_V4_MiSeq | -0.4 | -1.5 | 0.8 |  |  |  |  |  |  |  | 0.798 |
| Proteobacteria;unc | **RE-Model** | 0.1 | -0.4 | 0.5 | 7.4E-01 | 0 | 0.21 | 3.0 | 0.9 | 0.0 | 1.0 | 0.798 |
| Proteobacteria;unc | Burns_V56_MiSeq | 0.4 | -1.0 | 1.9 |  |  |  |  |  |  |  | 0.798 |
| Proteobacteria;unc | Chen_V13_454 | 0.2 | -1.3 | 1.6 |  |  |  |  |  |  |  | 0.798 |
| Proteobacteria;unc | Dejea_V35_454 | -0.4 | -2.1 | 1.3 |  |  |  |  |  |  |  | 0.798 |
| Proteobacteria;unc | Flemer_V34_MiSeq | 0.0 | -1.1 | 1.1 |  |  |  |  |  |  |  | 0.798 |
| Proteobacteria;unc | Kostic_V35_454 | 0.6 | -0.5 | 1.6 |  |  |  |  |  |  |  | 0.798 |
| Proteobacteria;unc | Marchesi_V13_454 | -0.2 | -2.3 | 2.0 |  |  |  |  |  |  |  | 0.798 |
| Proteobacteria;unc | katsu_V14_454 | -0.6 | -1.8 | 0.6 |  |  |  |  |  |  |  | 0.798 |
| Proteobacteria;unc | Zeller_V4_MiSeq | 0.3 | -0.9 | 1.4 |  |  |  |  |  |  |  | 0.798 |
| Firmicutes;Coprococcus | **RE-Model** | 0.0 | -0.4 | 0.4 | 9.2E-01 | 0 | 0.17 | 7.7 | 0.4 | 0.0 | 1.0 | 0.944 |
| Firmicutes;Coprococcus | Burns_V56_MiSeq | 0.0 | -1.5 | 1.4 |  |  |  |  |  |  |  | 0.944 |
| Firmicutes;Coprococcus | Chen_V13_454 | -1.0 | -2.5 | 0.5 |  |  |  |  |  |  |  | 0.944 |
| Firmicutes;Coprococcus | Dejea_V35_454 | 0.1 | -2.9 | 3.2 |  |  |  |  |  |  |  | 0.944 |
| Firmicutes;Coprococcus | Flemer_V34_MiSeq | -0.1 | -0.8 | 0.7 |  |  |  |  |  |  |  | 0.944 |
| Firmicutes;Coprococcus | Kostic_V35_454 | 1.8 | 0.1 | 3.4 |  |  |  |  |  |  |  | 0.944 |
| Firmicutes;Coprococcus | Marchesi_V13_454 | 1.2 | -1.5 | 3.9 |  |  |  |  |  |  |  | 0.944 |
| Firmicutes;Coprococcus | katsu_V14_454 | 0.3 | -1.2 | 1.9 |  |  |  |  |  |  |  | 0.944 |
| Firmicutes;Coprococcus | Zeller_V4_MiSeq | -0.2 | -1.0 | 0.6 |  |  |  |  |  |  |  | 0.944 |
| Firmicutes;[Eubacterium] | **RE-Model** | 0.0 | -0.5 | 0.5 | 9.7E-01 | 0 | 0.22 | 1.9 | 0.9 | 0.0 | 1.0 | 0.969 |
| Firmicutes;[Eubacterium] | Burns_V56_MiSeq | -0.2 | -1.6 | 1.3 |  |  |  |  |  |  |  | 0.969 |
| Firmicutes;[Eubacterium] | Chen_V13_454 | 0.4 | -1.1 | 1.9 |  |  |  |  |  |  |  | 0.969 |
| Firmicutes;[Eubacterium] | Flemer_V34_MiSeq | 0.1 | -0.7 | 0.8 |  |  |  |  |  |  |  | 0.969 |
| Firmicutes;[Eubacterium] | Kostic_V35_454 | -0.5 | -1.8 | 0.9 |  |  |  |  |  |  |  | 0.969 |
| Firmicutes;[Eubacterium] | Marchesi_V13_454 | 1.4 | -1.5 | 4.2 |  |  |  |  |  |  |  | 0.969 |
| Firmicutes;[Eubacterium] | Zeller_V4_MiSeq | -0.2 | -1.5 | 1.0 |  |  |  |  |  |  |  | 0.969 |
