# Supplementary material for: Re-purposing 16S rRNA gene sequence data from within case paired tumor biopsy and tumor-adjacent biopsy or fecal samples to identify microbial markers for colorectal cancer
Source: PLoS One. 2018 Nov 9;13(11):e0207002. doi: 10.1371/journal.pone.0207002 (PMC6226189; doi:10.1371/journal.pone.0207002)
Supplement: S3 Table — Taxonomy follows the convention of family, genus. Abbreviations for S3 Table: LogFC: Log2Fold Change, τ2: The (total) amount of heterogeneity among the true effects, SE: Standard error, QE: Test statistic for the test of (residual) heterogeneity from the full model, QEp: p-value associated with QE, I2: For a random-effects model, I2 estimates (in percent) how much of the total variability in the effect size estimates (which is composed of heterogeneity plus sampling variability) can be attributed to heterogeneity among the true effects, H2: estimates the ratio of the total amount of variability in the effect size estimates to the amount of sampling variability, FDR: False Discovery Rate, RE:Random Effects. (DOCX) [file pone.0207002.s007.docx]

| **Taxonomy** | **Study** | **LogFC** | **CILB** | **CIUB** | **p** | **tau** | **SE_Tau2** | **QE** | **QEp** | **I2** | **H2** | **FDR** |
| --- | --- | --- | --- | --- | --- | --- | --- | --- | --- | --- | --- | --- |
| Proteobacteria;Pseudomonas | **RE-Model** | 4.04 | 2.50 | 5.58 | 0.00 | 0.00 | 1.86 | 0.36 | 0.84 | 0.00 | 1.00 | 0.00 |
| Proteobacteria;Pseudomonas | Flemer_V34_MiSeq | 3.39 | 0.69 | 6.09 | 0.00 |  |  |  |  |  |  | 0.00 |
| Proteobacteria;Pseudomonas | Pascual_V13_454 | 4.26 | 1.63 | 6.89 | 0.00 |  |  |  |  |  |  | 0.00 |
| Proteobacteria;Pseudomonas | Chen_V13_454 | 4.49 | 1.64 | 7.35 | 0.00 |  |  |  |  |  |  | 0.00 |
| Firmicutes;Streptococcus | **RE-Model** | 1.95 | 0.89 | 3.02 | 0.00 | 0.00 | 0.92 | 0.49 | 0.78 | 0.00 | 1.00 | 0.01 |
| Firmicutes;Streptococcus | Flemer_V34_MiSeq | 2.09 | 0.51 | 3.67 | 0.00 |  |  |  |  |  |  | 0.01 |
| Firmicutes;Streptococcus | Pascual_V13_454 | 2.41 | 0.10 | 4.72 | 0.00 |  |  |  |  |  |  | 0.01 |
| Firmicutes;Streptococcus | Chen_V13_454 | 1.41 | -0.57 | 3.39 | 0.00 |  |  |  |  |  |  | 0.01 |
| Bacteroidetes;Porphyromonas | **RE-Model** | 2.30 | 0.72 | 3.87 | 0.00 | 0.00 | 1.96 | 0.43 | 0.81 | 0.00 | 1.00 | 0.06 |
| Bacteroidetes;Porphyromonas | Flemer_V34_MiSeq | 2.83 | 0.11 | 5.55 | 0.00 |  |  |  |  |  |  | 0.06 |
| Bacteroidetes;Porphyromonas | Pascual_V13_454 | 2.57 | -0.66 | 5.80 | 0.00 |  |  |  |  |  |  | 0.06 |
| Bacteroidetes;Porphyromonas | Chen_V13_454 | 1.66 | -0.88 | 4.20 | 0.00 |  |  |  |  |  |  | 0.06 |
| Firmicutes;Parvimonas | **RE-Model** | 2.02 | 0.31 | 3.74 | 0.02 | 1.61 | 2.50 | 6.44 | 0.09 | 53.47 | 2.15 | 0.20 |
| Firmicutes;Parvimonas | Flemer_V34_MiSeq | 2.15 | 0.02 | 4.29 | 0.02 |  |  |  |  |  |  | 0.20 |
| Firmicutes;Parvimonas | Pascual_V13_454 | 0.54 | -2.67 | 3.74 | 0.02 |  |  |  |  |  |  | 0.20 |
| Firmicutes;Parvimonas | Weir_V4_454 | 0.48 | -2.07 | 3.03 | 0.02 |  |  |  |  |  |  | 0.20 |
| Firmicutes;Parvimonas | Chen_V13_454 | 4.00 | 2.09 | 5.91 | 0.02 |  |  |  |  |  |  | 0.20 |
| Fusobacteria;Fusobacterium | **RE-Model** | 1.88 | 0.21 | 3.55 | 0.03 | 0.95 | 2.18 | 3.48 | 0.18 | 43.50 | 1.77 | 0.21 |
| Fusobacteria;Fusobacterium | Flemer_V34_MiSeq | 1.13 | -1.11 | 3.38 | 0.03 |  |  |  |  |  |  | 0.21 |
| Fusobacteria;Fusobacterium | Weir_V4_454 | 0.75 | -1.80 | 3.30 | 0.03 |  |  |  |  |  |  | 0.21 |
| Fusobacteria;Fusobacterium | Chen_V13_454 | 3.32 | 1.42 | 5.22 | 0.03 |  |  |  |  |  |  | 0.21 |
| Proteobacteria;unc | **RE-Model** | 1.31 | -0.17 | 2.78 | 0.08 | 0.00 | 1.70 | 0.66 | 0.72 | 0.00 | 1.00 | 0.40 |
| Proteobacteria;unc | Flemer_V34_MiSeq | 1.66 | -1.05 | 4.36 | 0.08 |  |  |  |  |  |  | 0.40 |
| Proteobacteria;unc | Pascual_V13_454 | 0.47 | -2.10 | 3.04 | 0.08 |  |  |  |  |  |  | 0.40 |
| Proteobacteria;unc | Weir_V4_454 | 1.83 | -0.72 | 4.37 | 0.08 |  |  |  |  |  |  | 0.40 |
| Firmicutes;unc | **RE-Model** | 1.22 | -0.16 | 2.60 | 0.08 | 0.00 | 1.62 | 2.62 | 0.45 | 0.00 | 1.00 | 0.40 |
| Firmicutes;unc | Flemer_V34_MiSeq | 2.47 | -0.26 | 5.19 | 0.08 |  |  |  |  |  |  | 0.40 |
| Firmicutes;unc | Pascual_V13_454 | 2.24 | -1.01 | 5.49 | 0.08 |  |  |  |  |  |  | 0.40 |
| Firmicutes;unc | Weir_V4_454 | 0.75 | -1.80 | 3.30 | 0.08 |  |  |  |  |  |  | 0.40 |
| Firmicutes;unc | Chen_V13_454 | -0.39 | -3.26 | 2.49 | 0.08 |  |  |  |  |  |  | 0.40 |
| Firmicutes;Ruminococcus | **RE-Model** | -0.79 | -1.63 | 0.06 | 0.07 | 0.00 | 0.61 | 2.88 | 0.41 | 0.00 | 1.00 | 0.40 |
| Firmicutes;Ruminococcus | Flemer_V34_MiSeq | -0.53 | -1.83 | 0.77 | 0.07 |  |  |  |  |  |  | 0.40 |
| Firmicutes;Ruminococcus | Pascual_V13_454 | -0.61 | -2.24 | 1.02 | 0.07 |  |  |  |  |  |  | 0.40 |
| Firmicutes;Ruminococcus | Weir_V4_454 | 0.03 | -2.44 | 2.50 | 0.07 |  |  |  |  |  |  | 0.40 |
| Firmicutes;Ruminococcus | Chen_V13_454 | -2.36 | -4.46 | -0.25 | 0.07 |  |  |  |  |  |  | 0.40 |
| Firmicutes;Phascolarctobacterium | **RE-Model** | -0.99 | -2.19 | 0.21 | 0.11 | 0.00 | 1.23 | 2.91 | 0.41 | 0.00 | 1.00 | 0.44 |
| Firmicutes;Phascolarctobacterium | Flemer_V34_MiSeq | 0.03 | -2.67 | 2.72 | 0.11 |  |  |  |  |  |  | 0.44 |
| Firmicutes;Phascolarctobacterium | Pascual_V13_454 | 0.06 | -2.31 | 2.44 | 0.11 |  |  |  |  |  |  | 0.44 |
| Firmicutes;Phascolarctobacterium | Weir_V4_454 | -1.55 | -3.67 | 0.57 | 0.11 |  |  |  |  |  |  | 0.44 |
| Firmicutes;Phascolarctobacterium | Chen_V13_454 | -2.58 | -5.40 | 0.24 | 0.11 |  |  |  |  |  |  | 0.44 |
| Firmicutes;Dialister | **RE-Model** | 1.33 | -0.33 | 2.98 | 0.12 | 0.65 | 2.18 | 2.63 | 0.27 | 29.58 | 1.42 | 0.44 |
| Firmicutes;Dialister | Flemer_V34_MiSeq | 2.43 | -0.28 | 5.13 | 0.12 |  |  |  |  |  |  | 0.44 |
| Firmicutes;Dialister | Pascual_V13_454 | 0.09 | -1.86 | 2.04 | 0.12 |  |  |  |  |  |  | 0.44 |
| Firmicutes;Dialister | Chen_V13_454 | 2.21 | -0.64 | 5.07 | 0.12 |  |  |  |  |  |  | 0.44 |
| Firmicutes;unc | Flemer_V34_MiSeq | -0.61 | -1.86 | 0.64 | 0.15 |  |  |  |  |  |  | 0.49 |
| Firmicutes;unc | Pascual_V13_454 | -0.63 | -2.14 | 0.89 | 0.15 |  |  |  |  |  |  | 0.49 |
| Firmicutes;unc | Weir_V4_454 | 0.75 | -1.77 | 3.27 | 0.15 |  |  |  |  |  |  | 0.49 |
| Firmicutes;unc | Chen_V13_454 | -2.74 | -4.26 | -1.22 | 0.15 |  |  |  |  |  |  | 0.49 |
| Firmicutes;unc | **RE-Model** | -0.96 | -2.26 | 0.34 | 0.15 | 1.06 | 1.43 | 7.79 | 0.05 | 62.59 | 2.67 | 0.49 |
| Firmicutes;Roseburia | Flemer_V34_MiSeq | -0.64 | -2.45 | 1.17 | 0.20 |  |  |  |  |  |  | 0.49 |
| Firmicutes;Roseburia | Pascual_V13_454 | 0.24 | -1.82 | 2.30 | 0.20 |  |  |  |  |  |  | 0.49 |
| Firmicutes;Roseburia | Chen_V13_454 | -2.29 | -4.11 | -0.47 | 0.20 |  |  |  |  |  |  | 0.49 |
| Firmicutes;Roseburia | **RE-Model** | -0.95 | -2.39 | 0.49 | 0.20 | 0.72 | 1.62 | 3.60 | 0.17 | 44.35 | 1.80 | 0.49 |
| Firmicutes;Faecalibacterium | Flemer_V34_MiSeq | -0.09 | -1.27 | 1.09 | 0.20 |  |  |  |  |  |  | 0.49 |
| Firmicutes;Faecalibacterium | Pascual_V13_454 | -0.76 | -2.26 | 0.73 | 0.20 |  |  |  |  |  |  | 0.49 |
| Firmicutes;Faecalibacterium | Weir_V4_454 | -0.59 | -3.13 | 1.95 | 0.20 |  |  |  |  |  |  | 0.49 |
| Firmicutes;Faecalibacterium | Chen_V13_454 | -1.09 | -2.99 | 0.80 | 0.20 |  |  |  |  |  |  | 0.49 |
| Firmicutes;Faecalibacterium | **RE-Model** | -0.50 | -1.28 | 0.27 | 0.20 | 0.00 | 0.51 | 1.01 | 0.80 | 0.00 | 1.00 | 0.49 |
| Proteobacteria;unc | Flemer_V34_MiSeq | 0.04 | -1.58 | 1.66 | 0.20 |  |  |  |  |  |  | 0.49 |
| Proteobacteria;unc | Pascual_V13_454 | 4.26 | 2.51 | 6.02 | 0.20 |  |  |  |  |  |  | 0.49 |
| Proteobacteria;unc | Weir_V4_454 | -0.65 | -3.11 | 1.80 | 0.20 |  |  |  |  |  |  | 0.49 |
| Proteobacteria;unc | Chen_V13_454 | 1.63 | 0.05 | 3.22 | 0.20 |  |  |  |  |  |  | 0.49 |
| Proteobacteria;unc | **RE-Model** | 1.38 | -0.73 | 3.50 | 0.20 | 3.77 | 3.79 | 16.24 | 0.00 | 82.32 | 5.66 | 0.49 |
| Firmicutes;Mogibacterium | **RE-Model** | 1.06 | -0.55 | 2.67 | 0.20 | 0.00 | 2.05 | 0.29 | 0.86 | 0.00 | 1.00 | 0.49 |
| Firmicutes;Mogibacterium | Pascual_V13_454 | 1.50 | -1.77 | 4.76 | 0.20 |  |  |  |  |  |  | 0.49 |
| Firmicutes;Mogibacterium | Weir_V4_454 | 1.29 | -1.26 | 3.84 | 0.20 |  |  |  |  |  |  | 0.49 |
| Firmicutes;Mogibacterium | Chen_V13_454 | 0.44 | -2.43 | 3.30 | 0.20 |  |  |  |  |  |  | 0.49 |
| Proteobacteria;Sutterella | **RE-Model** | 1.27 | -0.54 | 3.08 | 0.17 | 2.11 | 2.78 | 8.08 | 0.04 | 63.23 | 2.72 | 0.49 |
| Proteobacteria;Sutterella | Flemer_V34_MiSeq | 2.42 | 0.81 | 4.02 | 0.17 |  |  |  |  |  |  | 0.49 |
| Proteobacteria;Sutterella | Pascual_V13_454 | -0.07 | -2.29 | 2.14 | 0.17 |  |  |  |  |  |  | 0.49 |
| Proteobacteria;Sutterella | Weir_V4_454 | -0.65 | -3.12 | 1.83 | 0.17 |  |  |  |  |  |  | 0.49 |
| Proteobacteria;Sutterella | Chen_V13_454 | 3.41 | 0.54 | 6.28 | 0.17 |  |  |  |  |  |  | 0.49 |
| Firmicutes;unc | Flemer_V34_MiSeq | 1.82 | -0.89 | 4.54 | 0.34 |  |  |  |  |  |  | 0.63 |
| Firmicutes;unc | Pascual_V13_454 | 2.59 | -0.05 | 5.23 | 0.34 |  |  |  |  |  |  | 0.63 |
| Firmicutes;unc | Chen_V13_454 | -0.75 | -2.56 | 1.06 | 0.34 |  |  |  |  |  |  | 0.63 |
| Firmicutes;unc | **RE-Model** | 1.03 | -1.11 | 3.17 | 0.34 | 2.16 | 3.58 | 5.29 | 0.07 | 60.70 | 2.54 | 0.63 |
| Bacteroidetes;unc | Flemer_V34_MiSeq | 0.01 | -1.57 | 1.58 | 0.31 |  |  |  |  |  |  | 0.63 |
| Bacteroidetes;unc | Pascual_V13_454 | -2.45 | -4.18 | -0.71 | 0.31 |  |  |  |  |  |  | 0.63 |
| Bacteroidetes;unc | Weir_V4_454 | -0.01 | -2.46 | 2.43 | 0.31 |  |  |  |  |  |  | 0.63 |
| Bacteroidetes;unc | Chen_V13_454 | 0.10 | -2.79 | 2.99 | 0.31 |  |  |  |  |  |  | 0.63 |
| Bacteroidetes;unc | **RE-Model** | -0.72 | -2.10 | 0.66 | 0.31 | 0.90 | 1.62 | 5.44 | 0.14 | 46.25 | 1.86 | 0.63 |
| Firmicutes;Lachnospira | Flemer_V34_MiSeq | 0.79 | -0.89 | 2.47 | 0.32 |  |  |  |  |  |  | 0.63 |
| Firmicutes;Lachnospira | Pascual_V13_454 | -0.56 | -2.59 | 1.47 | 0.32 |  |  |  |  |  |  | 0.63 |
| Firmicutes;Lachnospira | Weir_V4_454 | -2.16 | -4.68 | 0.37 | 0.32 |  |  |  |  |  |  | 0.63 |
| Firmicutes;Lachnospira | Chen_V13_454 | -2.06 | -4.95 | 0.82 | 0.32 |  |  |  |  |  |  | 0.63 |
| Firmicutes;Lachnospira | **RE-Model** | -0.73 | -2.18 | 0.72 | 0.32 | 0.96 | 1.80 | 5.26 | 0.15 | 44.24 | 1.79 | 0.63 |
| Firmicutes;Lachnobacterium | **RE-Model** | -0.94 | -2.86 | 0.99 | 0.34 | 1.32 | 2.91 | 3.62 | 0.16 | 45.49 | 1.83 | 0.63 |
| Firmicutes;Lachnobacterium | Flemer_V34_MiSeq | 0.71 | -1.44 | 2.86 | 0.34 |  |  |  |  |  |  | 0.63 |
| Firmicutes;Lachnobacterium | Weir_V4_454 | -2.23 | -4.80 | 0.33 | 0.34 |  |  |  |  |  |  | 0.63 |
| Firmicutes;Lachnobacterium | Chen_V13_454 | -1.71 | -4.58 | 1.16 | 0.34 |  |  |  |  |  |  | 0.63 |
| Firmicutes;unc | **RE-Model** | 1.25 | -1.36 | 3.85 | 0.35 | 5.75 | 5.76 | 21.45 | 0.00 | 84.02 | 6.26 | 0.63 |
| Firmicutes;unc | Flemer_V34_MiSeq | -0.40 | -1.73 | 0.94 | 0.35 |  |  |  |  |  |  | 0.63 |
| Firmicutes;unc | Pascual_V13_454 | 0.74 | -2.40 | 3.88 | 0.35 |  |  |  |  |  |  | 0.63 |
| Firmicutes;unc | Weir_V4_454 | -0.39 | -2.95 | 2.17 | 0.35 |  |  |  |  |  |  | 0.63 |
| Firmicutes;unc | Chen_V13_454 | 4.94 | 2.96 | 6.91 | 0.35 |  |  |  |  |  |  | 0.63 |
| Firmicutes;[Eubacterium] | Flemer_V34_MiSeq | -0.96 | -3.13 | 1.20 | 0.42 |  |  |  |  |  |  | 0.72 |
| Firmicutes;[Eubacterium] | Pascual_V13_454 | 1.11 | -1.13 | 3.35 | 0.42 |  |  |  |  |  |  | 0.72 |
| Firmicutes;[Eubacterium] | Chen_V13_454 | -2.37 | -4.19 | -0.55 | 0.42 |  |  |  |  |  |  | 0.72 |
| Firmicutes;[Eubacterium] | **RE-Model** | -0.82 | -2.81 | 1.17 | 0.42 | 2.02 | 3.10 | 5.82 | 0.05 | 65.42 | 2.89 | 0.72 |
| Firmicutes;unc | Flemer_V34_MiSeq | -1.15 | -3.12 | 0.83 | 0.44 |  |  |  |  |  |  | 0.73 |
| Firmicutes;unc | Pascual_V13_454 | 0.09 | -2.95 | 3.13 | 0.44 |  |  |  |  |  |  | 0.73 |
| Firmicutes;unc | Weir_V4_454 | 0.00 | -2.40 | 2.41 | 0.44 |  |  |  |  |  |  | 0.73 |
| Firmicutes;unc | **RE-Model** | -0.53 | -1.86 | 0.81 | 0.44 | 0.00 | 1.44 | 0.75 | 0.69 | 0.00 | 1.00 | 0.73 |
| Firmicutes;Coprococcus | Flemer_V34_MiSeq | -0.63 | -1.90 | 0.64 | 0.62 |  |  |  |  |  |  | 0.94 |
| Firmicutes;Coprococcus | Pascual_V13_454 | 1.34 | -0.62 | 3.30 | 0.62 |  |  |  |  |  |  | 0.94 |
| Firmicutes;Coprococcus | Weir_V4_454 | 0.26 | -2.30 | 2.82 | 0.62 |  |  |  |  |  |  | 0.94 |
| Firmicutes;Coprococcus | Chen_V13_454 | -1.84 | -3.59 | -0.08 | 0.62 |  |  |  |  |  |  | 0.94 |
| Firmicutes;Coprococcus | **RE-Model** | -0.33 | -1.63 | 0.98 | 0.62 | 0.93 | 1.45 | 6.20 | 0.10 | 53.62 | 2.16 | 0.94 |
| Firmicutes;Blautia | Flemer_V34_MiSeq | -0.66 | -1.98 | 0.67 | 0.67 |  |  |  |  |  |  | 0.94 |
| Firmicutes;Blautia | Pascual_V13_454 | 2.07 | -0.28 | 4.42 | 0.67 |  |  |  |  |  |  | 0.94 |
| Firmicutes;Blautia | Weir_V4_454 | 0.40 | -2.15 | 2.95 | 0.67 |  |  |  |  |  |  | 0.94 |
| Firmicutes;Blautia | Chen_V13_454 | -3.12 | -4.67 | -1.57 | 0.67 |  |  |  |  |  |  | 0.94 |
| Firmicutes;Blautia | **RE-Model** | -0.46 | -2.61 | 1.68 | 0.67 | 3.82 | 3.90 | 15.77 | 0.00 | 82.12 | 5.59 | 0.94 |
| Firmicutes;Veillonella | Flemer_V34_MiSeq | -0.16 | -2.86 | 2.54 | 0.69 |  |  |  |  |  |  | 0.94 |
| Firmicutes;Veillonella | Pascual_V13_454 | -0.14 | -2.68 | 2.40 | 0.69 |  |  |  |  |  |  | 0.94 |
| Firmicutes;Veillonella | Weir_V4_454 | -1.43 | -3.95 | 1.08 | 0.69 |  |  |  |  |  |  | 0.94 |
| Firmicutes;Veillonella | Chen_V13_454 | 3.69 | 0.84 | 6.54 | 0.69 |  |  |  |  |  |  | 0.94 |
| Firmicutes;Veillonella | **RE-Model** | 0.42 | -1.69 | 2.54 | 0.69 | 2.89 | 3.79 | 7.81 | 0.05 | 62.26 | 2.65 | 0.94 |
| Firmicutes;Oscillospira | Flemer_V34_MiSeq | -1.05 | -2.54 | 0.44 | 0.68 |  |  |  |  |  |  | 0.94 |
| Firmicutes;Oscillospira | Pascual_V13_454 | 0.09 | -1.84 | 2.03 | 0.68 |  |  |  |  |  |  | 0.94 |
| Firmicutes;Oscillospira | Weir_V4_454 | -0.59 | -3.13 | 1.95 | 0.68 |  |  |  |  |  |  | 0.94 |
| Firmicutes;Oscillospira | Chen_V13_454 | 1.49 | -0.93 | 3.90 | 0.68 |  |  |  |  |  |  | 0.94 |
| Firmicutes;Oscillospira | **RE-Model** | -0.22 | -1.28 | 0.84 | 0.68 | 0.19 | 0.97 | 3.41 | 0.33 | 15.53 | 1.18 | 0.94 |
| Firmicutes;Gemella | **RE-Model** | 0.92 | -3.15 | 4.98 | 0.66 | 11.01 | 12.90 | 14.17 | 0.00 | 85.87 | 7.08 | 0.94 |
| Firmicutes;Gemella | Flemer_V34_MiSeq | 0.71 | -2.01 | 3.44 | 0.66 |  |  |  |  |  |  | 0.94 |
| Firmicutes;Gemella | Pascual_V13_454 | -2.77 | -6.03 | 0.49 | 0.66 |  |  |  |  |  |  | 0.94 |
| Firmicutes;Gemella | Chen_V13_454 | 4.41 | 2.22 | 6.60 | 0.66 |  |  |  |  |  |  | 0.94 |
| Firmicutes;unc | Flemer_V34_MiSeq | -0.15 | -1.50 | 1.19 | 0.72 |  |  |  |  |  |  | 0.95 |
| Firmicutes;unc | Pascual_V13_454 | 0.69 | -0.88 | 2.26 | 0.72 |  |  |  |  |  |  | 0.95 |
| Firmicutes;unc | Weir_V4_454 | -0.41 | -2.70 | 1.87 | 0.72 |  |  |  |  |  |  | 0.95 |
| Firmicutes;unc | Chen_V13_454 | -0.63 | -1.99 | 0.72 | 0.72 |  |  |  |  |  |  | 0.95 |
| Firmicutes;unc | **RE-Model** | -0.14 | -0.89 | 0.61 | 0.72 | 0.00 | 0.47 | 1.71 | 0.63 | 0.00 | 1.00 | 0.95 |
| Bacteroidetes;Parabacteroides | Flemer_V34_MiSeq | 0.10 | -1.33 | 1.54 | 0.97 |  |  |  |  |  |  | 0.97 |
| Bacteroidetes;Parabacteroides | Pascual_V13_454 | -0.39 | -2.18 | 1.40 | 0.97 |  |  |  |  |  |  | 0.97 |
| Bacteroidetes;Parabacteroides | Weir_V4_454 | 1.44 | -0.97 | 3.84 | 0.97 |  |  |  |  |  |  | 0.97 |
| Bacteroidetes;Parabacteroides | Chen_V13_454 | -0.62 | -2.41 | 1.17 | 0.97 |  |  |  |  |  |  | 0.97 |
| Bacteroidetes;Parabacteroides | **RE-Model** | -0.01 | -0.88 | 0.85 | 0.97 | 0.00 | 0.64 | 2.12 | 0.55 | 0.00 | 1.00 | 0.97 |
| Firmicutes;Anaerostipes | Flemer_V34_MiSeq | -0.26 | -1.89 | 1.36 | 0.90 |  |  |  |  |  |  | 0.97 |
| Firmicutes;Anaerostipes | Pascual_V13_454 | 1.46 | -1.41 | 4.33 | 0.90 |  |  |  |  |  |  | 0.97 |
| Firmicutes;Anaerostipes | Weir_V4_454 | 0.53 | -2.01 | 3.08 | 0.90 |  |  |  |  |  |  | 0.97 |
| Firmicutes;Anaerostipes | Chen_V13_454 | -1.78 | -4.65 | 1.09 | 0.90 |  |  |  |  |  |  | 0.97 |
| Firmicutes;Anaerostipes | **RE-Model** | -0.07 | -1.19 | 1.04 | 0.90 | 0.00 | 1.11 | 2.84 | 0.42 | 0.00 | 1.00 | 0.97 |
| Firmicutes;unc | Flemer_V34_MiSeq | -0.07 | -1.24 | 1.09 | 0.78 |  |  |  |  |  |  | 0.97 |
| Firmicutes;unc | Pascual_V13_454 | 0.49 | -0.93 | 1.92 | 0.78 |  |  |  |  |  |  | 0.97 |
| Firmicutes;unc | Weir_V4_454 | 0.99 | -1.24 | 3.22 | 0.78 |  |  |  |  |  |  | 0.97 |
| Firmicutes;unc | Chen_V13_454 | -1.38 | -2.73 | -0.03 | 0.78 |  |  |  |  |  |  | 0.97 |
| Firmicutes;unc | **RE-Model** | -0.13 | -1.08 | 0.81 | 0.78 | 0.39 | 0.76 | 5.20 | 0.16 | 42.47 | 1.74 | 0.97 |
| Firmicutes;Dorea | Flemer_V34_MiSeq | -0.17 | -1.36 | 1.01 | 0.87 |  |  |  |  |  |  | 0.97 |
| Firmicutes;Dorea | Pascual_V13_454 | 2.45 | 0.19 | 4.71 | 0.87 |  |  |  |  |  |  | 0.97 |
| Firmicutes;Dorea | Weir_V4_454 | -0.86 | -3.40 | 1.68 | 0.87 |  |  |  |  |  |  | 0.97 |
| Firmicutes;Dorea | Chen_V13_454 | -0.52 | -1.97 | 0.94 | 0.87 |  |  |  |  |  |  | 0.97 |
| Firmicutes;Dorea | **RE-Model** | 0.10 | -1.07 | 1.26 | 0.87 | 0.64 | 1.15 | 5.76 | 0.12 | 46.44 | 1.87 | 0.97 |
| Bacteroidetes;unc | Flemer_V34_MiSeq | -0.56 | -1.93 | 0.82 | 0.88 |  |  |  |  |  |  | 0.97 |
| Bacteroidetes;unc | Pascual_V13_454 | 0.61 | -1.06 | 2.29 | 0.88 |  |  |  |  |  |  | 0.97 |
| Bacteroidetes;unc | Weir_V4_454 | -0.70 | -3.17 | 1.76 | 0.88 |  |  |  |  |  |  | 0.97 |
| Bacteroidetes;unc | Chen_V13_454 | 0.50 | -1.68 | 2.68 | 0.88 |  |  |  |  |  |  | 0.97 |
| Bacteroidetes;unc | **RE-Model** | -0.07 | -0.94 | 0.80 | 0.88 | 0.00 | 0.65 | 1.71 | 0.63 | 0.00 | 1.00 | 0.97 |
| Bacteroidetes;Bacteroides | Flemer_V34_MiSeq | -0.58 | -1.91 | 0.74 | 0.96 |  |  |  |  |  |  | 0.97 |
| Bacteroidetes;Bacteroides | Pascual_V13_454 | -1.84 | -3.31 | -0.38 | 0.96 |  |  |  |  |  |  | 0.97 |
| Bacteroidetes;Bacteroides | Weir_V4_454 | -0.59 | -2.79 | 1.60 | 0.96 |  |  |  |  |  |  | 0.97 |
| Bacteroidetes;Bacteroides | Chen_V13_454 | 2.80 | 1.22 | 4.37 | 0.96 |  |  |  |  |  |  | 0.97 |
| Bacteroidetes;Bacteroides | **RE-Model** | -0.05 | -2.04 | 1.93 | 0.96 | 3.44 | 3.36 | 19.94 | 0.00 | 84.56 | 6.48 | 0.97 |
| Firmicutes;[Ruminococcus] | Flemer_V34_MiSeq | 0.01 | -1.12 | 1.15 | 0.92 |  |  |  |  |  |  | 0.97 |
| Firmicutes;[Ruminococcus] | Pascual_V13_454 | 3.04 | 0.04 | 6.04 | 0.92 |  |  |  |  |  |  | 0.97 |
| Firmicutes;[Ruminococcus] | Weir_V4_454 | 0.57 | -1.97 | 3.12 | 0.92 |  |  |  |  |  |  | 0.97 |
| Firmicutes;[Ruminococcus] | Chen_V13_454 | -2.13 | -3.64 | -0.62 | 0.92 |  |  |  |  |  |  | 0.97 |
| Firmicutes;[Ruminococcus] | **RE-Model** | 0.11 | -1.84 | 2.05 | 0.92 | 2.89 | 3.21 | 11.56 | 0.01 | 77.94 | 4.53 | 0.97 |
| Actinobacteria;Collinsella | Flemer_V34_MiSeq | -0.57 | -2.87 | 1.73 | 0.97 |  |  |  |  |  |  | 0.97 |
| Actinobacteria;Collinsella | Weir_V4_454 | 0.99 | -1.55 | 3.54 | 0.97 |  |  |  |  |  |  | 0.97 |
| Actinobacteria;Collinsella | Chen_V13_454 | -0.48 | -3.34 | 2.37 | 0.97 |  |  |  |  |  |  | 0.97 |
| Actinobacteria;Collinsella | **RE-Model** | -0.03 | -1.46 | 1.41 | 0.97 | 0.00 | 1.62 | 0.96 | 0.62 | 0.00 | 1.00 | 0.97 |
| Bacteroidetes;Prevotella | Flemer_V34_MiSeq | -0.03 | -1.60 | 1.54 | 0.89 |  |  |  |  |  |  | 0.97 |
| Bacteroidetes;Prevotella | Pascual_V13_454 | -3.29 | -5.20 | -1.38 | 0.89 |  |  |  |  |  |  | 0.97 |
| Bacteroidetes;Prevotella | Weir_V4_454 | 0.87 | -1.69 | 3.43 | 0.89 |  |  |  |  |  |  | 0.97 |
| Bacteroidetes;Prevotella | Chen_V13_454 | 2.02 | -0.09 | 4.14 | 0.89 |  |  |  |  |  |  | 0.97 |
| Bacteroidetes;Prevotella | **RE-Model** | -0.16 | -2.41 | 2.09 | 0.89 | 4.24 | 4.31 | 15.59 | 0.00 | 81.10 | 5.29 | 0.97 |
