# Supplementary material for: Re-purposing 16S rRNA gene sequence data from within case paired tumor biopsy and tumor-adjacent biopsy or fecal samples to identify microbial markers for colorectal cancer
Source: PLoS One. 2018 Nov 9;13(11):e0207002. doi: 10.1371/journal.pone.0207002 (PMC6226189; doi:10.1371/journal.pone.0207002)
Supplement: S4 Table — (DOCX) [file pone.0207002.s008.docx]

**Supplementary Table 4. Genera present in both fecal and mucosal samples, only in fecal samples and only in biopsy samples.**

| **Phylum** | **Family** | **Genus** | **Occurrence** |
| --- | --- | --- | --- |
| p__Proteobacteria | f__Enterobacteriaceae | g__ | Fecal and Biopsy |
| p__Bacteroidetes | f__Bacteroidaceae | g__Bacteroides | Fecal and Biopsy |
| p__Firmicutes | f__[Tissierellaceae] | g__Parvimonas | Fecal and Biopsy |
| p__Firmicutes | f__Lachnospiraceae | g__Roseburia | Fecal and Biopsy |
| p__Firmicutes | f__Ruminococcaceae | g__Ruminococcus | Fecal and Biopsy |
| p__Fusobacteria | f__Fusobacteriaceae | g__Fusobacterium | Fecal and Biopsy |
| p__Firmicutes | f__Ruminococcaceae | g__Oscillospira | Fecal and Biopsy |
| p__Proteobacteria | f__Alcaligenaceae | g__Sutterella | Fecal and Biopsy |
| p__Firmicutes | f__Streptococcaceae | g__Streptococcus | Fecal and Biopsy |
| p__Proteobacteria | f__Enterobacteriaceae | g__Morganella | Fecal and Biopsy |
| p__Firmicutes | f__Lachnospiraceae | g__Dorea | Fecal and Biopsy |
| p__Firmicutes | f__Lachnospiraceae | g__Blautia | Fecal and Biopsy |
| p__Firmicutes | f__Veillonellaceae | g__Selenomonas | Fecal and Biopsy |
| p__Firmicutes | f__Clostridiaceae | g__Clostridium | Fecal and Biopsy |
| p__Bacteroidetes | f__[Odoribacteraceae] | g__Odoribacter | Fecal and Biopsy |
| p__Firmicutes | f__Peptostreptococcaceae | g__Peptostreptococcus | Fecal and Biopsy |
| p__Verrucomicrobia | f__Verrucomicrobiaceae | g__Akkermansia | Fecal and Biopsy |
| p__Firmicutes | f__Veillonellaceae | g__Phascolarctobacterium | Fecal and Biopsy |
| p__Proteobacteria | f__Pasteurellaceae | g__Haemophilus | Fecal and Biopsy |
| p__Firmicutes | f__Ruminococcaceae | g__Faecalibacterium | Fecal and Biopsy |
| p__Bacteroidetes | f__Prevotellaceae | g__Prevotella | Fecal and Biopsy |
| p__Actinobacteria | f__Propionibacteriaceae | g__Propionibacterium | Fecal and Biopsy |
| p__Firmicutes | f__Lachnospiraceae | g__Anaerostipes | Fecal only |
| p__Proteobacteria | f__Shewanellaceae | g__Shewanella | Fecal only |
| p__Proteobacteria | f__Moraxellaceae | g__Acinetobacter | Fecal only |
| p__Proteobacteria | f__Halomonadaceae | g__Halomonas | Fecal only |
| p__Proteobacteria | f__Enterobacteriaceae | g__Klebsiella | Fecal only |
| p__Euryarchaeota | f__Methanobacteriaceae | g__Methanobrevibacter | Fecal only |
| p__Firmicutes | f__Enterococcaceae | g__Enterococcus | Fecal only |
| p__Firmicutes | f__Veillonellaceae | g__Succiniclasticum | Fecal only |
| p__Actinobacteria | f__Corynebacteriaceae | g__Corynebacterium | Biopsy only |
| p__Proteobacteria | f__Sphingomonadaceae | g__Sphingomonas | Biopsy only |
| p__Proteobacteria | f__Enterobacteriaceae | g__Escherichia | Biopsy only |
| p__Firmicutes | f__Erysipelotrichaceae | g__Allobaculum | Biopsy only |
| p__Firmicutes | f__Leuconostocaceae | g__Weissella | Biopsy only |
| p__Firmicutes | f__Erysipelotrichaceae | g__Catenibacterium | Biopsy only |
